# Examining the Acceptability and Effectiveness of a Self-Directed, Web-Based Resource for Stress and Coping in University: Randomized Controlled Trial

**DOI:** 10.2196/74205

**Published:** 2026-01-23

**Authors:** Bilun Naz Böke, Jessica Mettler, Laurianne Bastien, Sohyun Cho, Nancy Heath

**Affiliations:** 1 Department of Educational and Counselling Psychology, Faculty of Education, McGill University, 3700 McTavish Street, Montreal, QC, H3A1X1, Canada, 1 5143984242; 2 Department of Psychology, Concordia University, Montreal, QC, Canada

**Keywords:** stress, coping, self-directed programming, web-based intervention, university students

## Abstract

**Background:**

University students face high levels of stress with limited support for coping and well-being. Campus mental health services are increasingly using digital resources to support students’ stress management and coping capacity. However, the effectiveness of providing this support through web-based, self-directed means remains unclear.

**Objective:**

Using a randomized controlled design, this study examined the acceptability and effectiveness of a self-directed, web-based resource containing evidence-based strategies for stress management and healthy coping for university students. The study additionally explored the potential benefits of screening and directing students to personalized resources aligned with their needs.

**Methods:**

Participants consisted of 242 university students (193/242, 79.9% women; mean age 21.15 years) assigned to one of 3 groups (ie, automatically directed to personalized resources, nondirected, and waitlist comparison). They completed pre, post (4 wk), and follow-up (8 wk) measures for stress, coping, and well-being. The resource groups also completed acceptability measures at 2, 4, and 8 weeks after the web-based resource access.

**Results:**

Results indicate high acceptability, reflecting students’ satisfaction with the resource. Furthermore, significant decreases in stress and unhealthy coping, as well as significant increases in coping self-efficacy and healthy coping in the resource groups relative to the comparison group, were found. Interestingly, the directed approach showed no added benefit over nondirected resource access.

**Conclusions:**

In summary, this study demonstrates the acceptability and effectiveness of a self-directed digital resource platform as a viable support option for university student stress and coping.

## Introduction

### Background

University students consistently report high levels of stress and psychological distress and identify these as key factors that negatively impact their academic performance and engagement with their studies [1-4]. Supporting students in effectively coping with stress and distress is of critical importance to facilitate learning and development in university environments. To that end, technology-based approaches to delivering stress-management and well-being supports to university students have proliferated on campuses as supplemental means of supporting student stress management, coping capacity, and well-being [5]. Indeed, resources for students’ self-directed use, such as websites, apps, or on-demand workshops, are increasingly popular given their benefits in improving access to support as well as the potential for reaching students who may be reluctant to seek other forms of mental health support or are on waiting lists for more specialized services [6]. In addition, the provision of resources for addressing stress and enhancing coping capacity is aligned with the recently proposed health theory of coping, which calls for enhancing the availability of evidence-based healthy coping strategies [7]. However, investigation into the acceptability, and even more critically, the effectiveness of digital, self-directed resources for nonclinical stress management and healthy coping support is limited. Thus, this study sought to explore the acceptability and effectiveness of a self-directed, web-based resource for enhancing students’ stress management and coping capacity. Furthermore, the study also examined whether there would be any added benefit of screening students to assess stress and coping needs and then directing them to specific resources to match their needs for stress management and healthy coping support.

### University Student Stress and Coping

University students’ mental health and well-being have been a growing concern within higher education research and practice for many decades [8-10]. The most frequently identified factors impacting academic performance in recent population-level surveys (n=54,204) include stress (43.7%), anxiety (37.3%), depression (27.5%), and sleep difficulties (25.9%) [1]. Within Canada, university students (n=11,322) identified the same factors: stress (51.5%), anxiety (43.3%), depression (30.4%), and sleep difficulties (31.9%), as having had a negative impact on their academic performance over the past year [2]. For those pursuing a university education, this time in their lives often corresponds with their developmental transition to adulthood [11]. Coined in research literature as emerging adulthood, this developmental period is distinct from adulthood conceptually and as a subjective experience [12-15].

Emerging adulthood is a challenging yet unique time of exploration and settling into adult roles, often characterized as a time of feeling in-between [12,13]. While the transition to adulthood brings increased autonomy and responsibility, this period is also marked by instability across multiple life domains, including relationships, living arrangements, employment, and identity development. Navigating these changes can heighten vulnerability, stress, difficulties with coping, and mental health challenges. Notably, emerging adulthood is associated with elevated rates of engagement in risky and unhealthy coping behaviors in response to stress and distress [16-18]. For example, Böke et al [16] found that university students reporting higher stress were more likely to engage in substance use as a coping strategy. Conversely, research has shown that skill-based approaches to coping, such as problem-focused coping, defined as actively addressing the source of stress through problem-solving or planning, can buffer the negative impact of stress on well-being [19]. Taken together, there is a clear need to enhance access to evidence-based strategies and tools to support students in effectively managing stress and enhancing their capacity to cope with distress [19-22].

To enhance coping capacity among university students, understanding their decision-making processes in coping with stress is imperative. The health theory of coping offers a comprehensive framework for conceptualizing how students cope with stress and distress [7]. Stallman’s health theory of coping considers all coping responses as adaptive, emphasizing their short-term efficacy in alleviating momentary stress or distress and further classifies coping responses into healthy and unhealthy coping behaviors based on the likelihood of adverse consequences. The theory presents a hierarchical model delineating coping responses across intensities, directly corresponding to the intensity of experienced stress or distress [7,19]. Low levels of stress or distress prompt low-intensity coping, encompassing both healthy (eg, positive self-talk, mindfulness, abdominal breathing) and unhealthy (eg, negative self-talk, cognitive rumination, suppression) responses. As distress intensifies, coping responses escalate, where higher intensity healthy strategies may include engaging in distracting activities, relaxation, physical exercise, or seeking social/professional support, while unhealthy responses may involve self-isolation, emotional eating, self-harm, substance use, or suicidality [7]. Acknowledging this hierarchical progression is pivotal in designing student support programs tailored to promote the availability of and engagement in evidence-based healthy coping behaviors.

### Supporting Stress-Management and Building Coping Capacity

To date, efforts aimed at improving student mental health and well-being in university settings have included a wide variety of interventions targeting stress [23], depression [24], anxiety [6], resilience [25], and general mental health and well-being [26]. Increasingly, technology-based and digital tools (eg, websites, apps, chatbots, on-demand programming) are used with several systematic and meta-analytic reviews emphasizing the promise of the technology-based approach for improving key outcomes [5,26-28]. Furthermore, emerging research demonstrates the promise of sharing resources for students’ self-directed use at their own pace and discretion [29-32].

For example, Fischer et al [33] demonstrated that self-directed interventions were effective in improving well-being and reducing stress, depression, and anxiety among both the general population and clinical samples when compared with active and inactive controls. This is supported by 2 meta-analytic reviews reporting significant effects of self-guided interventions for improving depressive symptoms in general population samples [34,35]. Among university students, a meta-analysis by Bolinski et al [29] found online mental health interventions (the majority were self-directed) to be effective for reducing anxiety and depression, although only a small and nonsignificant effect was reported for academic performance. In addition, Chung et al [36] examined the effectiveness of a university-wide, self-directed online mindfulness and well-being intervention and found improvements across stress, well-being, and mindfulness outcomes for those who engaged with the intervention over a duration of 3 or more weeks.

Self-directed or self-administered digital resources have the potential to serve as supplementary support for students and offer several advantages. First, they have the potential to reach those who may not access face-to-face services, who may not meet clinical criteria for specialized treatments, or are on waitlists for services, thus broadening access to evidence-based strategies and supports [30,35,37,38]. Second, the self-guided format is supportive of student autonomy and confidentiality as individuals can choose when, where, and how to access information and make use of resources most aligned with their individual needs [37]. Last, the web-based presentation of information and evidence-based strategies and techniques allows for a cost-effective, low-intensity, and adaptable (ie, possibility to update or change based on contextual needs) means to supplement existing mental health and well-being services on campus [6,39,40]. Furthermore, studies suggest that this modality is welcomed in universities [37,41] where up to 70% of students in a sample of 1224 indicated interest in self-guided mental health supports [42].

### Issues With Supporting University Student Stress-Management and Healthy Coping

It should be noted that digital stress-management tools that are often developed for general adult or workplace populations and retroactively adapted for university students were found not to adequately address the developmental and contextual realities of this population [37]. As highlighted by Fleischmann et al [37], students face a unique combination of stressors at a precarious developmental transition, including academic and adjustment pressures, identity development, and unstable life circumstances that differ from those of working adults. Their findings underscore that students value support options that are specifically tailored to the academic context and their unique developmental needs while offering flexibility around fluctuating needs. Moreover, students report a desire for resources that reflect their lived experiences and offer personalized guidance and recommendations [37]. This suggests a need to include university students in the development of resources that are personalized to their unique needs, which may in turn enhance students’ engagement with such resources. Despite emerging evidence of effectiveness for using digital, self-directed approaches to student support, research examining the effectiveness and acceptability of this approach is in its infancy. In addition, it is unclear to what extent digital, self-directed programming and resources are integrated into the university setting and used beyond their initial effectiveness trials [30]. Notably, even when interventions and programs for student mental health and well-being are shown to be effective, they are often only shared with students through the universities’ health and wellness center, relying on students to proactively seek help to access these services. This poses a challenge because research consistently shows that university students exhibit low levels of help-seeking, leading to the underuse of many services and resources despite a high demand [43,44]. Additionally, earlier studies exploring means to support students’ stress and coping have focused on addressing one aspect of stress or coping, such as mindfulness for stress, or breathing exercises for managing anxiety [45]. This signals a need for broader resources covering a wider array of topics and coping strategies to build coping capacity. Taken together, there is an urgent need to explore alternative approaches for resource delivery that facilitate students’ universal and ongoing access to self-directed support options to comprehensively address stress and coping needs.

A persistent problem in university and a barrier to students’ access to support is low rates of help-seeking, where stigma around mental health difficulties is considered to be a major contributor to students’ reluctance to seek support [44,46]. Emerging research suggests that perceived mental health stigma can also contribute to students’ responses to the format and modality of stress-management and well-being support delivery [47]. Specifically, Cho et al’s [47] intervention study found that students’ perceived mental health stigma did not impact their sustained satisfaction with a self-directed modality (ie, an infographic presenting evidence-based strategies for stress management and well-being), while it negatively impacted their sustained satisfaction with a live digital workshop presenting the same information with the presence of a facilitator. Beyond stigma, students may prefer digital, self-directed supports for several reasons, including concerns about confidentiality, social anxiety, and wanting to avoid social interactions focused on a topic that they would like to keep private. Overall, proactively connecting students to available resources is therefore an important consideration to navigate the effect of mental health stigma and other barriers on students’ help-seeking behavior and promote their engagement with support services. One suggested solution for this is the use of brief screening measures to identify students’ levels of need for support and recommend existing resources aligned with their personal needs [6,48,49]. Indeed, this approach has shown promise in clinical contexts as part of suicide prevention efforts in universities [49,50]. For example, in a large-scale study, Hasking et al [49] found that the use of a multivariable screener for suicidal risk followed by referral to a stepped telehealth intervention significantly increased resource use among university students classified as having the greatest need for intervention. Whether screening and tailoring resource recommendations can also promote students’ engagement with, and use of, low-intensity stress-management and healthy coping resources in a nonclinical context remains to be explored.

Moreover, there is a need to consider students’ uptake of stress-management and healthy coping strategies presented in self-directed resources. In a systematic review of prevention programs for stress, depression, and anxiety in university contexts, which included self-administered programming, Rith-Najarian et al [51] found inconsistencies in the assessment and reporting of information on uptake and adherence. Specifically, only 57% of the studies included in the review presented any information on adherence or completion, which prevented the authors from including adherence as a factor within their analyses [51]. A later study examining the effectiveness of a self-directed mindfulness intervention delivered over 12 weeks reported that students’ access to the program modules peaked during the first 3 weeks, declined steeply over weeks 3 to 7, and then stabilized with a small increase in the final week 12 [36]. Overall, the authors reported that 58.7% of their total sample (n=833) did not access the mindfulness program at all over the duration of the semester-long study [36]. Assessing and reporting uptake or use of the provided resource is of particular importance in studies examining self-directed modalities where use can fluctuate over time and where the proportion of zero-uptake may be elevated. Furthermore, rates of uptake or use may influence the accuracy of effectiveness findings, and additional research is needed to better understand the relation between program uptake/adherence and outcomes of effectiveness [51].

### This Study

In summary, despite the rapid proliferation of digital self-guided resources for university students, research examining the effectiveness of this approach for improving stress and coping is still in its infancy. Further research is needed to address gaps and deepen our understanding of what works best and how in the area of supporting university students’ stress management and coping capacity [6,51]. Thus, using a randomized-controlled design, this study sought to examine the acceptability and effectiveness of a web-based, self-directed resource for university students containing evidence-based strategies for stress management and healthy coping. In addition, this study examined whether there would be any added benefit of using a screening approach to direct students to personalized resources aligned with their identified needs. Participants were randomly assigned to one of 3 groups: directed to personalized resources aligned with needs, nondirected but received all resources, or a waitlist comparison. Main outcomes assessed were participant ratings of acceptability, stress, coping (coping self-efficacy and coping behaviors), and well-being over time.

Specifically, the first objective (1) was to examine potential group differences (directed and nondirected resource groups only) in students’ acceptability of the web-based resource over time. It was hypothesized that (H1) acceptability would be higher in the directed group when compared with the nondirected group over time. The second objective (2) was to examine the effectiveness of the digital self-directed resources in terms of group differences (directed, nondirected, and comparison) on outcome measures (ie, stress, coping, and well-being) and in terms of differences in scores over time between baseline, post, and follow-up measures. It is hypothesized (H2a) that the directed group will show greater improvements across stress, coping, and well-being outcomes over time than both the nondirected group and the comparison group. It is also hypothesized (H2b) that the nondirected group will show significant improvements across study outcomes relative to the comparison group. Last, the third objective (3) was to examine the effectiveness of the overall web-based, self-directed resource in terms of group differences (resource group; merged directed and nondirected vs the comparison group) on outcome measures and in terms of change in scores over time between baseline, post, and follow-up measures (ie, stress, coping, and well-being). It is hypothesized (H3) that the resource group will show significant improvements across study outcomes in relation to the comparison group.

## Methods

### Ethical Considerations

All procedures in this study were approved by the Research Ethics Board of McGill University (number 21-10-040). Informed consent was obtained prior to study participation; all participants were informed that they could choose to withdraw or end their participation in the study at any point without penalty or prejudice. Participant data have been aggregated for the purposes of data analysis and publication to respect privacy and confidentiality. Study participants received compensation of CAD $50 (US $36.40) via e-transfer for their participation. This study was registered as a randomized controlled trial on ClinicalTrials.gov (NCT07086001), and the associated study checklist is provided in Checklist 1.

### Participants

Eligibility criteria included (1) being enrolled as a student at the university where the study took place and (2) being 18 years of age or older. Participants consisted of 242 university students recruited across a large university (193/242, 79.9% women; mean age 21.15). Participants were randomly assigned to one of 3 study groups (directed: 65/81 [80.5%] women, mean age 21.31; nondirected: 66/81, 81.5% women, mean age 21.07; comparison: 62/80, 77.8% women, mean age 21.06).

### Resource Development and Content

The development of the web-based resource examined in this study was informed by 3 key foundational frameworks, namely, the health theory of coping [7], the theory of emerging adulthood [12,13], and Stepped Care 2.0 (SC2.0) [52,53]. Specifically, the health theory of coping provides a conceptual framework depicting university students’ approaches to coping with stress and distress across a hierarchical spectrum where the intensity of the coping behavior is proportional to the intensity of experienced distress [7]. The theory of emerging adulthood and research describing general characteristics of this developmental period were instrumental in informing the topics and content developed and presented within the digital resource [12,13]. Last, SC2.0 presents a stepped, hierarchical framework for the organization of campus mental health care and services across incremental steps of intensity [52,53]. The resource tested within this study aligns with the lower intensity steps within SC2.0, and the framework has influenced and informed the screening and referral to personalized resources (ie, directed vs nondirected) model tested within this study. In addition, resource development followed a collaborative approach with a large team of university students (undergraduate and graduate), researchers, and university mental health service professionals consulting at each project stage (eg, conceptualization, material development, implementation, and data collection).

Overall, the theoretical foundations described above, the environmental scan of best practices in digital resource creation, as well as consultations with the project team informed the scope of topics and content areas to create research-informed resources with evidence-based strategies and tips. For example, students particularly requested resources for topics such as dealing with breakups, managing household responsibilities, managing stress around finances, setting and maintaining boundaries, and building social connections, among others. A priori, it was determined that resources would be presented in several multimedia formats (ie, text, audio, video, interactive infographic) to account for the diversity of preferences. In sum, there were over 50 different resources developed to highlight evidence-based strategies for healthy coping, addressing a broad scope of topics relevant to emerging adult university students in a demanding academic context. All resources were grouped in 5 main categories: managing stress, which presented strategies for coping with everyday stressors and enhancing emotion regulation capacity; enhancing performance, focused on skills around enhancing academic performance such as motivation, time management, and responding to academic setbacks; adulting addressed skills around the transitional life stage such as career exploration, relational changes (eg, breakups), and financial management; socializing offered guidance on building and maintaining meaningful social connections and dealing with loneliness; and well-being presented strategies that support psychological resilience such as gratitude, mindfulness, and self-awareness. Additionally, a psychoeducation and information-based section titled Understanding was created to share general statistics and information pertaining to university student stress, mental health, and well-being. The website also presented an additional resources section to connect students to, and encourage their use of, other services and resources they are eligible for at the university, in the local community, and through other websites and apps.

Importantly, given the web-based nature of the resource, accessibility of digital content was a key consideration throughout development and implementation. Consistent with Web Content Accessibility Guidelines 2.0 [54], features across content included accessible font styles and sizes, high-contrast color schemes, screen reader compatibility, plain language, and a mobile-optimized version of the website to support diverse user needs.

### Procedure

#### Overview

Participants who expressed interest in participating in the study were asked to complete a brief digital demographics survey to facilitate their random assignment into the 3 different conditions within the study; namely, directed to resources based on reported need in the screening questionnaire (Group 1: directed), nondirected sharing of all resources (Group 2: nondirected), and waitlist comparison (Group 3: comparison). Participants were randomly assigned to the 3 conditions by the study lead author using IBM SPSS Statistics (version 23) tools, where participant IDs were randomly organized into 3 separate groups. Blinding was not deemed necessary as the study was conducted entirely digitally and directing to the resource was automated. Responses to the demographic questionnaire were used to ensure comparable samples across the different conditions in terms of participants’ age, gender, and program of study. Following random assignment to the different conditions, all participants were asked to complete the baseline measures and the screening questionnaire (described in the measures section below). Although all participants were asked to complete the brief screening questionnaire, only those in the directed group subsequently received personalized instruction on how to use the resources and strategies provided in the digital resource.

#### Group 1 (Directed)

Immediately following the completion of the baseline survey, Group 1 was given access to the website presenting a collection of stress-management, motivation, healthy coping, well-being, and socializing resources. Additionally, based on their answers to the brief screener, Group 1 was directed to one of 3 unique pages on the website based on their responses on the screening questionnaire, demonstrating low, moderate, or high need for support around stress and coping. The directing process was automated using a scoring algorithm within the survey platform used in this study (ie, Qualtrics). Details on the screening questions, algorithm, and cut-off scores are provided in the Multimedia Appendix 1.

#### Group 2 (Nondirected)

Participants in Group 2 followed the same procedure as Group 1; however, they did not receive any personalized instruction and were simply directed to the home page of the website containing resources.

#### Group 3 (Comparison)

Participants in Group 3 constituted the waitlist comparison group. As such, they did not have access to any of the strategies hosted on the website during the data collection phase of the study. Participants in Group 3 were asked to complete web-based surveys identical to those completed by Groups 1 and 2. Although Group 3 did not have access to the strategies during the project, the full web-based resource was shared with the comparison group at the end of data collection.

In terms of data collection timeline, all groups completed measures (detailed in the next section) regarding their stress, coping, and well-being at the start of the study (baseline: T1), 4 weeks after the start of the study (post: T2), and 8 weeks following the start of the study (follow-up: T3). In addition, participants in Groups 1 and 2 completed a brief check-in to assess resource acceptability 2 weeks after baseline, which is when the resources were initially shared with participants.

### Measures

#### Screening

The purpose of this screening questionnaire was to assess students’ varying levels of need for support around stress, distress, coping, self-efficacy, loneliness, and social support to enable the directing of Group 1 (directed) to resources that match their need for stress-management and healthy coping support. This screener consisted of a 24-item researcher-designed measure comprised of a mix of single items assessing coping behaviors, financial stress, and access to community, as well as short versions of standardized measures that have been shown to be associated with university students’ overall adjustment and well-being including, perceived stress [55], coping self-efficacy [56], loneliness [57], social support [58], and social connectedness [59]. Participants in the directed group were categorized as indicating high, moderate, or low need for stress-management and coping support based on their scores on the researcher-developed screening questionnaire and were subsequently directed to unique pages of the web-based resource. The scoring and categorization algorithm is described in the Multimedia Appendix 1. In brief, cut-off scores were set as the top/bottom 15th percentile score within the sample for each section of the screener; ie, stress and coping behaviors (general stress), perceived stress, coping self-efficacy (intrapersonal), loneliness, social support, and social connectedness (interpersonal). Participants with scores meeting or exceeding the cut-off across the general stress, interpersonal, and intrapersonal sections were categorized as having a high need for support. Participants with scores meeting or exceeding the cut-off in at least one section were categorized as having moderate need for support. Last, those with scores below the cut-off across all sections of the screener were categorized as having low need for support. The distribution of high, moderate, and low need categories is provided in Table 1. In terms of the pages they were directed to, those scoring in the high need category were directed to comprehensive resources for stress and coping support in the community, crisis lines, as well as specific help-seeking strategies (note that no participants in this study scored in the high need category across groups; thus, they did not receive the direction described above, and the implications are discussed in the Results and Discussion sections). Those indicating moderate need for support were directed to the full web-based resource and encouraged to use the presented strategies. Last, those indicating low need for support were directed to the understanding section of the website to provide further information around stress and coping, as well as a list of evidence-based stress-management and healthy coping strategies for their quick use in the event they feel a need.

**Table 1.** Participant demographic information and screener scores: full sample (n=212) and the subsample of participants (n=177) who reported at least some use of the digital resource*.*

	Full sample			Subsample		
	Directed	Nondirected	Comparison	Directed	Nondirected	Comparison
Age (years), mean (SD)	21.22 (2.68)	21.17 (3.13)	20.81 (2.19)	20.70 (1.79)	21.04 (3.21)	20.81 (2.19)
Gender, n (%)						
Woman	60 (83.3)	54 (81.8)	59 (79.7)	44 (81.5)	41 (83.7)	59 (79.7)
Man	11 (15.3)	9 (13.6)	14 (18.9)	10 (18.5)	7 (14.3)	14 (18.9)
Nonbinary	0 (0)	3 (4.5)	1 (1.4)	0 (0)	1 (2)	1 (1.4)
Prefer not to say	1 (1.4)	0 (0)	0 (0)	0 (0)	0 (0)	0 (0)
Faculty of study, n (%)						
Agriculture & Environmental Science	5 (6.9)	6 (9.1)	5 (6.8)	5 (9.3)	3 (6.1)	5 (6.8)
Arts	18 (25)	17 (25.8)	21 (28.4)	12 (22.2)	13 (26.5)	21 (28.4)
Continuing Studies	1 (1.4)	0 (0)	0 (0)	1 (1.9)	0 (0)	0 (0)
Education	1 (1.4)	1 (1.5)	4 (5.4)	1 (1.9)	1 (2)	4 (5.4)
Engineering	1 (1.4)	0 (0)	0 (0)	0 (0)	0 (0)	0 (0)
Law	5 (6.9)	4 (6.1)	2 (2.7)	3 (5.6)	2 (4.1)	2 (2.7)
Management	19 (26.4)	24 (36.4)	24 (32.4)	18 (33.3)	20 (40.8)	24 (32.4)
Medicine	1 (1.4)	2 (3)	3 (4.1)	1 (1.9)	2 (4.1)	3 (4.1)
Music	1 (1.4)	0 (0)	0 (0)	1 (1.9)	0 (0)	0 (0)
Nursing	3 (4.2)	1 (1.5)	0 (0)	2 (3.7)	1 (2)	0 (0)
Science	13 (18.1)	6 (9.1)	11 (14.9)	8 (14.8)	4 (8.2)	11 (14.9)
Other^a^	4 (5.6)	5 (7.6)	4 (5.4)	2 (3.7)	3 (6.1)	4 (5.4)
Screener score^b^, n (%)						
Low need	44 (61.1)	42 (63.6)	50 (67.6)	34 (63)	29 (59.2)	50 (67.6)
Moderate need	28 (38.9)	24 (36.4)	24 (32.4)	20 (37)	20 (40.8)	24 (32.4)
High need	0 (0)	0 (0)	0 (0)	0 (0)	0 (0)	0 (0)

a The category of “Other” for Faculty of Study included those in cross-faculty programs (eg, Arts & Science).

b The scoring algorithm for the screener to determine low, moderate, and high need categories is provided in Multimedia Appendix 1.

#### Acceptability

Participants’ ratings of the acceptability of the resources and strategies shared were assessed using a researcher-developed measure aligned with the Kirkpatrick New World Model for program evaluation [60]. Specifically, a total of 11 items assessed participants’ (1) overall satisfaction with the resource (8 items; eg, “I found the website useful for me”; “The strategies presented in the website helped me better understand how to manage my stress and improve my wellness”; “I found that the website presented valuable strategies and techniques” rated on a 4-point Likert scale; 1=“strongly disagree” to 4=“strongly agree”), (2) frequency of actual and planned use of strategies (2 items; ie, “Over the past two weeks, how often did you use the strategies presented on the website?*”* and *“*Over the coming weeks, how often do you plan to use the strategies presented on the website?*”* Rated on a 4-point Likert scale; 1=“every day” to 4=“never”) as well as (3) a single item to rate perceived impact for their well-being (ie, “Over the past two weeks, how would you rate the impact of the strategies presented on the website on your well-being?*”* Rated on a 4-point Likert scale; 1=“no impact” to 4=“high impact”). Scores were summed for the first part of the measure depicting satisfaction (ie, items 1‐8), and the remaining items (actual and planned strategy use, impact on well-being) were analyzed as single-item responses. Internal consistency of the satisfaction subscale was good in this study (*α*=.88, .85, .87 at 2 weeks post baseline, T2, and T3, respectively). The complete version of the acceptability questionnaire is presented in the Multimedia Appendix 1.

#### Stress

Participants’ perceived level of general stress was assessed using the 10-item version of the Perceived Stress Scale (PSS) [61]. This measure is a widely used self-report measure of adults’ perception of stress. The items ask participants to indicate their experience of stress and the degree to which life situations are stressful on a 5-point scale; 0=“never” to 4=“very often.” Items include statements such as “In the last two weeks, how often have you felt difficulties were piling up so high that you could not overcome them?” and “In the past two weeks, how often have you felt nervous and stressed?” Higher scores on the PSS represent greater perceived stress. The PSS has adequate internal reliability, construct validity, and predictive validity with reports of psychological and physical symptoms and the use of health services [55]. Although the original measure asks participants to report perceived stress over the last month, the measure was adapted in this study for consistency of timeline across measures; therefore, the prompt was adapted to ask that participants report their perceived stress over the past 2 weeks. Descriptive statistics for the PSS-10 in this study (Tables2  3) were deemed comparable to those reported among other university student samples (mean 19.79, SD 6.37) [62]. The internal consistency of the PSS in this study was good (*α*=.83, .84, .85 at T1, T2, T3, respectively).

**Table 2.** Series of 3 (group: active, passive, comparison) × 3 (time: baseline, post, follow-up) mixed design ANOVAs for mental health and well-being outcomes among a subsample of participants who reported using the strategies presented in the digital resource (n=177)*.*

Outcome	Time point	Directed (n=54), mean (SD)	Nondirected (n=49), mean (SD)	Comparison (n=74), mean (SD)
Stress				
Int^a^^,^ ^b^ (*F*_3.807, 331.190_=2.571, *P*=.04, η_p_^2^=.029)	Baseline	22.09 (5.68)	21.96 (6.24)	21.92 (6.20)
MET^c^^,^ ^d^ (*F*_1.903, 331.190_=6.613, *P*=.002, η_p_^2^=.037)	Post	20.77 (6.36)	19.70 (5.77)	21.40 (6.73)
MEG^e^ (*F*_2, 174_=0.770, *P*=.46, η_p_^2^=.009)	Follow-up	19.78 (5.46)	20.53 (6.35)	22.19 (6.92)
Coping self-efficacy				
Int (*F*_4, 348_=2.395, *P*=.052, η_p_^2^=.027)	Baseline	143.10 (36.91)	136.66 (37.58)	143.78 (42.04)
MET^d^ (*F*_2, 348_=8.993, *P*<.001, η_p_^2^=.049)	Post	147.54 (42.91)	146.70 (37.24)	145.25 (45.41)
MEG (*F*_2, 174_=.0325, *P*=.70, η_p_^2^=.004)	Follow-up	158.55 (38.30)	151.35 (39.45)	144.81 (43.78)
Healthy coping				
Int (*F*_4, 348_=1.978, *P*=.098, η_p_^2^=.022)	Baseline	12.00 (3.94)	11.71 (3.11)	12.02 (3.56)
MET^d^ (*F*_2, 348_=15.962, *P*<.001, η_p_^2^=.084)	Post	13.12 (3.56)	12.66 (3.31)	12.34 (3.54)
MEG (*F*_2, 174_=0.688, *P*=.50, η_p_^2^=.008)	Follow-up	13.69 (3.90)	13.46 (3.17)	12.46 (4.07)
Unhealthy coping				
Int^b^ (*F*_3.697, 321.674_=2.937, *P*=.02, η_p_^2^=.033)	Baseline	9.43 (9.43)	9.86 (3.15)	9.63 (3.93)
MET^d^ (*F*_1.849, 321.674_=9.603, *P*<.001, η_p_^2^=.052)	Post	8.67 (8.67)	9.52 (3.01)	9.54 (3.57)
MEG (*F*_2, 174_=1.235, *P*=.29, η_p_^2^=.014)	Follow-up	8.20 (8.20)	8.19 (2.98)	9.57 (3.60)
Well-being				
Int (*F*_4, 348_=0.611, *P*=.65, η_p_^2^=.007)	Baseline	3.09 (0.65)	3.16 (0.59)	3.24 (0.64)
MET (*F*_2, 348_=0.762, *P*=.46, η_p_^2^=.004)	Post	3.17 (0.66)	3.23 (0.59)	3.24 (0.77)
MEG (*F*_1, 174_=0.169, *P*=.84, η_p_^2^=.002)	Follow-up	3.18 (0.66)	3.19 (0.67)	3.19 (0.72)

a Int: Interaction.

b *P*<.05.

c MET: main effect of time.

d *P*<.001; Bonferroni correction (*P*=.05/3=.0167) was used at the level of main effects to account for multiple comparisons.

e MEG: main effect of group.

**Table 3.** Series of 2 (group: resource, comparison) × 3 (time: baseline, post, follow-up) mixed design ANOVAs for mental health and well-being outcomes after merging the directed and nondirected groups into a single resource group (n=177)*.*

Outcome	Time point	Resource group, mean (SD)	Comparison, mean (SD)
Stress			
Int^a^^,^ ^b^ (*F*_1.911, 334.382_=3.597, *P*=.03, η_p_^2^=.020)	Baseline	22.03 (5.92)	21.92 (6.20)
MET^b^^,^ ^c^ (*F*_1.911, 334.382_=4.230, *P*=.02, η_p_^2^=.024)	Post	20.26 (6.08)	21.40 (6.73)
MEG^d^ (*F*_1, 175_=1.530, *P*=.22, η_p_^2^=.009)	Follow-up	20.14 (5.89)	22.19 (6.92)
Coping self-efficacy			
Int^b^ (*F*_2, 350_=4.196, *P*=.02, η_p_^2^=.023)	Baseline	140.04 (37.19)	143.78 (42.04)
MET^e^ (*F*_1.943, 339.997_=5.448, *P*=.005, η_p_^2^=.030)	Post	147.14 (40.12)	145.25 (45.41)
MEG (*F*_1, 175_=0.257, *P*=.61, η_p_^2^=.001)	Follow-up	155.12 (38.83)	144.81 (43.78)
Healthy coping			
Int^b^ (*F*_2, 350_=3.894, *P*=.02, η_p_^2^=.022)	Baseline	11.86 (3.55)	12.02 (3.56)
MET^e^ (*F*_2, 350_=11.259, *P*<.001, η_p_^2^=.060)	Post	12.90 (3.43)	12.34 (3.54)
MEG (*F*_1, 175_=1.109, *P*=.29, η_p_^2^=.006)	Follow-up	13.58 (3.56)	12.46 (4.07)
Unhealthy coping			
Int^b^ (*F*_1.854, 324.520_=4.784, *P*=.01, η_p_^2^=.027)	Baseline	9.63 (3.11)	9.63 (3.93)
MET^e^ (*F*_1.854, 324.520_=5.532, *P*=.005, η_p_^2^=.031)	Post	9.08 (2.96)	9.54 (3.57)
MEG^d^ (*F*_1, 175_=1.921, *P*=.17, η_p_^2^=.011)	Follow-up	8.20 (3.13)	9.57 (3.60)
Well-being			
Int^a^ (*F*_2, 350_=.989, *P*=.37, η_p_^2^=.006)	Baseline	3.13 (0.62)	3.24 (0.64)
MET^c^ (*F*_2, 350_=.513, *P*=.60, η_p_^2^=.003)	Post	3.20 (0.63)	3.24 (0.77)
MEG^d^ (*F*_1, 175_=.367, *P*=.55, η_p_^2^=.002)	Follow-up	3.18 (0.66)	3.19 (0.72)

a Int: Interaction.

b *P*<.05.

c MET: main effect of time.

d MEG: main effect of group.

e *P*<.001; Bonferroni correction (*P*=.05/3=.0167) was used at the level of main effects to account for multiple comparisons.

#### Coping

Participants’ belief in their ability to cope with general difficulty and distress was assessed using the Coping Self-Efficacy Scale (CSE) [56]. The CSE is a measure of one’s confidence in effectively engaging in coping behaviors in the face of challenges. There are 26 items and 3 subscales within the CSE; namely, problem-focused coping (12 items), emotion-focused coping (9 items), and social support (5 items). Participants are asked to rate their confidence in their ability to perform the listed coping behaviors (eg, “find solutions to your most difficult problems,” “see things from the other person’s point of view during a heated argument”) on an 11-point Likert scale; 0=“cannot do at all” to 10=“certainly can do.” Higher scores on the CSE represent greater belief in one’s own ability to cope with difficulty. The CSE demonstrated negative correlations with perceived stress, burnout [56], and emotion regulation difficulties [63]. Conversely, the CSE is positively correlated with optimism [56]. In this study, the prompt for this measure was adapted to ask participants about their confidence in their ability to perform the listed coping behaviors, specifically over the past 2 weeks, and the internal consistency of the full CSE was excellent (*α*=.93, .95, and .95, at T1, T2, and T3, respectively).

In addition, the Coping Index (CI) [64] was used to assess students’ engagement in healthy and unhealthy coping behaviors over the duration of the study. The CI is a 20-item measure of engagement with healthy (10 items) and unhealthy (10 items) coping behaviors, which are aligned with the health theory of coping framework [7]. The measure consists of items that list common healthy and unhealthy coping behaviors, such as “talk things over with family or friends,” “do relaxing activities,” or “have negative self-talk.” Participants are asked to indicate how often they engage in each behavior listed when they feel stressed or distressed on a 4-point Likert scale (0= “I don’t do this at all” to 3= “I do this most of the time”). Higher scores on the healthy coping subscale indicate greater frequency of engagement in healthy coping behaviors; similarly, higher scores on the unhealthy coping subscale indicate greater frequency of engagement in unhealthy coping in response to stress or distress. This measure has been found to have satisfactory test-retest reliability in previous studies (*α*=.71) [65]. In this study, internal consistency of the healthy coping subscale was poor (*α*=.57, .57, .64 at T1, T2, T3, respectively), and the unhealthy coping subscale was also poor (*α*=.53, .53, .58 at T1, T2, T3, respectively). This is expected and deemed borderline acceptable for research purposes [66], given that the items within the subscales of the CI assess unique coping behaviors that may not necessarily have high agreement between them.

#### Well-Being

Well-being was assessed using the Warwick-Edinburgh Mental Well-Being Scale (WEMWBS) [67]. This measure consists of 14 positively worded items assessing overall subjective well-being. Participants are asked to rate statements such as “I’ve been feeling good about myself” according to their experience over the past 2 weeks on a 5-point Likert scale (1=“none of the time” to 5=“all of the time”). A higher WEMWBS score represents a higher level of mental well-being. The WEMWBS has demonstrated good internal consistency within university students (*α*=.89) and general population samples (*α*=.91). Test-retest reliability after a one-week delay was also high (*α*=.83) [67]. The internal consistency of the WEMWBS in this study was excellent (*α*=.91, .92, .93 at T1, T2, T3, respectively).

#### Data Analytic Plan

The overarching purpose of the study was to examine the acceptability and the effectiveness of a self-guided digital resource for university student stress, coping, and well-being outcomes. Preliminary analyses (ie, a one-way ANOVA, chi-square tests) were conducted to ensure comparability of the 3 study groups on demographic variables such as age, gender, and faculty of study at baseline. Given the importance of actual engagement with the digital resource for the accurate assessment of acceptability [51], the analyses of acceptability (Objective 1) were conducted both within the full study sample and a subsample of participants consisting of those who reported using the resources at least sometimes across all timepoints. Preliminary descriptive statistics were computed to examine students’ satisfaction with the digital resource, their reported and intended use of strategies, and the perceived impact of using the strategies on their well-being among both the directed and nondirected groups. Group differences in satisfaction and strategy use ratings were examined using a series of 2-way mixed design ANOVAs to examine the effects of condition (directed vs nondirected delivery of resources) and time (baseline, post, and follow-up) on student ratings of satisfaction and strategy use, as well as the reported impact of strategy use on their well-being. Across all analyses, the Bonferroni correction was used across at the level of main effects, simple main effects, and pairwise comparisons to account for multiple comparisons.

Notably, there were a total of 35 (14.46% of the total sample) participants (mean age 22.00, SD 3.48; 78.9% women) in the resource groups that reported never using the presented digital resource and strategies. In a resource evaluation study, those who were assigned to a resource group but chose not to engage with the resource cannot comment on the resources, nor would we expect the resources to effect a change, and this data may interfere with the accurate evaluation of the effectiveness of the resources. Compared with students who reported using the strategies (n=103; n=177 when including 74 participants from the comparison group), those who reported never using the strategies (n=35) were not significantly different on any of the study variables (stress, coping, and well-being) at baseline. Therefore, those who reported never using the strategies were excluded from the subsequent analyses, which were only conducted among the subsample of participants who reported using the resource at least sometimes across the 3 timepoints (directed: n=54, mean age 20.70, SD 1.79, 81.5% women; nondirected: n=49, mean age 21.04, SD 3.208, 83.7% women; comparison: n=74, mean age 20.81, SD 2.19, 79.7% women). The criterion of “at least sometimes” was used to ensure that participants had some degree of exposure to the web-based resource, as prior research suggests that even minimal engagement is necessary for participants to provide informed feedback on effectiveness and acceptability [51]. This threshold was therefore chosen to distinguish between no use and at least some use of the web-based resource being tested.

Thus, for the accurate assessment of effectiveness (Objective 2), analyses were restricted to the subsample consisting of participants who reported at least some use of the digital resource across the study timeline. A series of 3 (condition: directed, nondirected, waitlist comparison) × 3 (time: baseline, post, follow-up) mixed-design ANOVAs were used to examine potential changes in stress, coping, and well-being over time.

Last for objective 3, which sought to examine the overall effectiveness of the digital resource against a business-as-usual comparison group, the directed and nondirected groups were merged into one “resource group*”* to facilitate this analysis. A series of 2 (condition: resource group, waitlist comparison) × 3 (time: baseline, post, follow-up) mixed-design ANOVAs were used to examine potential changes in stress, coping, and well-being over time. Across all analyses, follow-up examination of main effects and simple main effects of group and time was conducted to locate any observed differences by group or over time. Bonferroni corrections were used across main effects and simple main effects analyses to account for multiple comparisons. IBM SPSS (version 23; IBM Corp) was used for all analyses in this study.

## Results

### Preliminary Analyses

**Figure 1.**
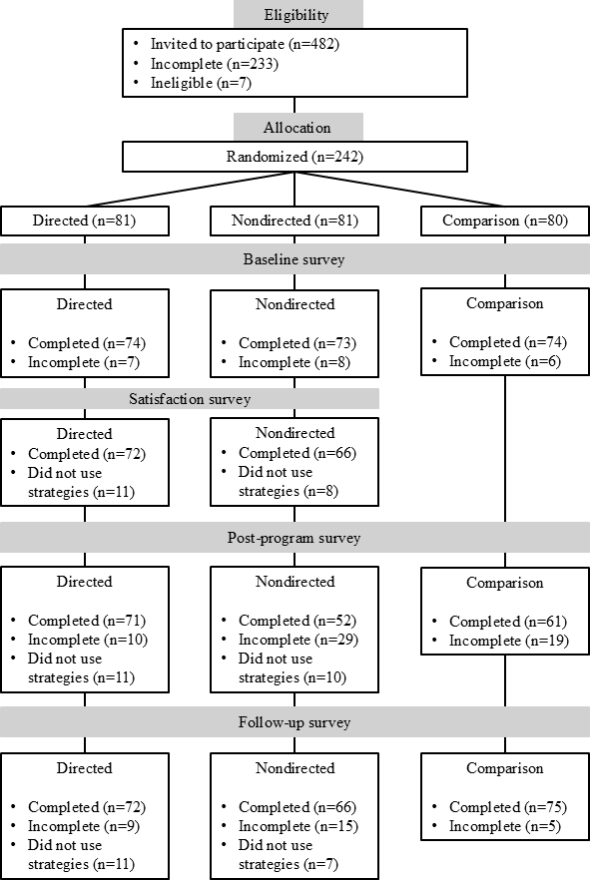
Participant flow diagram.

Participants were randomly assigned to the directed, nondirected, and comparison groups following their completion of the demographic questionnaire. A one-way ANOVA revealed no differences based on age across the study groups, *F*_2,229_=0.139, *P*=.87. Two chi-square tests of independence revealed no associations across the groups by gender, *χ*^2^_6_=5.9, *P*=.44, or faculty of study, *χ*^2^_22_=18.3, *P*=.69. Thus, the efficacy of the randomization and comparability of the study groups was supported. A total of 19 participants were excluded from all analyses, given that most of their digital surveys were incomplete. Missing values analyses demonstrated less than 5% of missing data within each timepoint and group, which were imputed using the Expectation Maximization method. There were 4 univariate outliers identified (*z*>|3.29|) which were winsorized for data conservation. Thus, the final study sample consisted of 212 participants (mean age 21.06, SD 2.67, 81.6% women). As noted above, this study also considered the subsample of participants who reported at least some use of the strategies shared on the web-based resource (Figure 1 displays the participant flow diagram). Demographic characteristics and screener scores of both the full sample and the subsample of participants are displayed in Table 1. Interestingly, participants’ scores on the screener indicate either low or moderate need for stress-management and healthy coping support, with no participant scores signaling high need. The proportion of low versus moderate need, as indicated by screener scores, was comparable across all study groups (directed, nondirected, and comparison).

### Objective 1: Acceptability of the Self-Directed Digital Resource as Assessed by Group Differences (Directed Vs Nondirected) Over Time (Baseline to Follow-up) on Overall Resource Satisfaction, Actual and Planned Strategy Use, and Perceived Impact on Well-Being

Participants in both the directed (Group 1) and nondirected (Group 2) conditions rated the digital resource very highly, with specific ratings across the acceptability questionnaire for each group across time depicted in the Multimedia Appendix 1. Overall, participants indicated that the strategies presented in the digital resource were valuable (90% and 92% agreed in Groups 1 and 2, respectively), presented in an engaging manner (83% and 86% in Groups 1 and 2, respectively), and easy to understand (93% and 94% in Groups 1 and 2, respectively). Similarly, up to 83% of those in the directed group and 79% of those in the nondirected group agreed that the strategies presented helped them better understand how to manage their stress and improve their wellness.

A 2-way mixed design ANOVA to assess group differences over time for overall satisfaction with the digital resource (sum score of acceptability items 1 to 8) revealed no significant group by time interaction; *F*_1.764,206.390_=0.015, *P*=.98, *η_p_^2^*=.000 (Table 4). Similarly, no interactions were found for strategy use, *F*_1.793,208.039_=.204, *P*=.79, *η_p_^2^*=.002; planned strategy use, *F*_2,232_=1.554, *P*=.21, *η_p_^2^*=.013; and perceived impact of strategy use on well-being; *F*_2,234_=0.067, *P*=.93, *η_p_^2^*=.001. Analyses of main effects revealed no significant changes in strategy use over time using the Bonferroni correction; *F*_1.793,208.039_=3.576, *P*=.03, *η_p_^2^*=.030. Similarly, there was no significant main effect of time (MET) for participants’ ratings of perceived impact of strategy use on their well-being; *F*_2,234_=3.694, *P*=.03, *η_p_^2^*p2=.031. Thus, the first hypothesis (H1), expecting higher overall acceptability (satisfaction, strategy use, and impact on well-being) within the directed group, was not supported, with both groups reporting comparably high levels of acceptability for the digital resource.

**Table 4.** Series of 2 (group: directed, nondirected) × 3 (time: pre, post, follow-up) mixed design ANOVAs for acceptability of web-based resource.

Sample and outcome	Time point	Directed, mean (SD)	Nondirected, mean (SD)
Full sample (directed: n=54; nondirected: n=49)			
Satisfaction sum			
Int^a^ (*F*_1.764, 206.390_=0.015, *P*=.98, η_p_^2^=.000)	Pre	23.60 (4.57)	23.91 (3.02)
MET^b^ (*F*_1.764, 206.390_=1.332, *P*=.27, η_p_^2^=.011)	Post	23.52 (3.85)	23.77 (3.13)
MEG^c^ (*F*_1, 117_=0.176, *P*=.68, η_p_^2^=.002)	Follow-up	24.03 (4.00)	24.23 (3.87)
Strategy use			
Int^a^ (*F*_1.793, 208.039_=0.204, *P*=.79, η_p_^2^=.002)	Pre	3.10 (0.50)	3.04 (0.51)
MET^d^ (*F*_1.793, 208.039_=3.576, *P*=.03, η_p_^2^=.030	Post	3.05 (0.52)	3.05 (0.52)
MEG^c^ (*F*_1, 116_=0.097, *P*=.76, η_p_^2^=.001)	Follow-up	2.95 (0.68)	2.93 (0.54)
Planned strategy use			
Int^a^ (*F*_2, 232_=1.554, *P*=0.21, η_p_^2^=.013)	Pre	2.76 (0.56)	2.61 (0.65)
MET^b^ (*F*_2, 232_=1.696, *P*=.19, η_p_^2^=.014)	Post	2.61 (0.66)	2.66 (0.58)
MEG^c^ (*F*_1, 116_=0.479, *P*=.49, η_p_^2^=.004)	Follow-up	2.79 (0.68)	2.70 (0.63)
Impact on well-being			
Int^a^ (*F*_2, 234_=0.067, *P*=.93, η_p_^2^=.001)	Pre	2.44 (0.82)	2.39 (0.76)
MET^b^ (*F*_2, 234_=3.694, *P*=.03, η_p_^2^=.031)	Post	2.54 (0.78)	2.48 (0.81)
MEG^c^ (*F*_1, 117_=0.317, *P*=.58, η_p_^2^=.003)	Follow-up	2.65 (0.83)	2.55 (0.74)
Subsample (directed: n=46; nondirected: n=41)			
Satisfaction sum			
Int^a^ (*F*_1.696, 144.162_=0.266, *P*=.73, η_p_^2^=.003)	Pre	24.57 (4.08)	24.83 (2.62)
MET^b^ (*F*_1.696, 144.162_=1.894, *P*=.16, η_p_^2^=.022)	Post	24.41 (3.12)	24.63 (2.89)
MEG^c^ *(F*_1, 85_=0.032, *P*=.86, η_p_^2^=.000)	Follow-up	25.26 (3.14)	25.07 (2.92)
Strategy use			
Int^a^ (*F*_1.610, 135.257_=0.479, *P*=.58, η_p_^2^=.006)	Pre	2.89 (0.31)	2.85 (0.36)
MET^b^ (*F*_1.610, 135.257_=3.447, *P*=.04, η_p_^2^=.039)	Post	2.85 (0.36)	2.85 (0.36)
MEG^c^ (*F*_1, 84_=.009, *P*=.92, η_p_^2^=.000)	Follow-up	2.72 (0.54)	2.78 (0.42)
Planned strategy use			
Int^a^ (*F*_2, 168_=1.810, *P*=.17, η_p_^2^=.021)	Pre	2.62 (0.49)	2.44 (0.63)
MET^b^ (*F*_2, 168_=1.343, *P*=.26, η_p_^2^=.016	Post	2.49 (0.66)	2.54 (0.55)
MEG^c^ (*F*_1, 84_=0.108, *P*=.74, η_p_^2^=.001)	Follow-up	2.60 (0.62)	2.63 (0.62)
Impact on well-being			
Int^a^ (*F*_2, 170_=0.665, *P*=.51, η_p_^2^=.008)	Pre	2.70 (0.66)	2.66 (0.57)
MET^b^ (*F*_2, 170_=5.299, *P*=.01, η_p_^2^=.059)	Post	2.83 (0.57)	2.78 (0.57)
MEG^c^ (*F*_1, 85_=0.811, *P*=.37, η_p_^2^=.009)	Follow-up	2.98 (0.49)	2.80 (0.51)

a Int: Interaction.

b MET: main effect of time.

c MEG: main effect of group.

d *P*<.05, Bonferroni correction (*P*=.05/2=.025) was used at the level of main effects to account for multiple comparisons.

**Figure 2.**
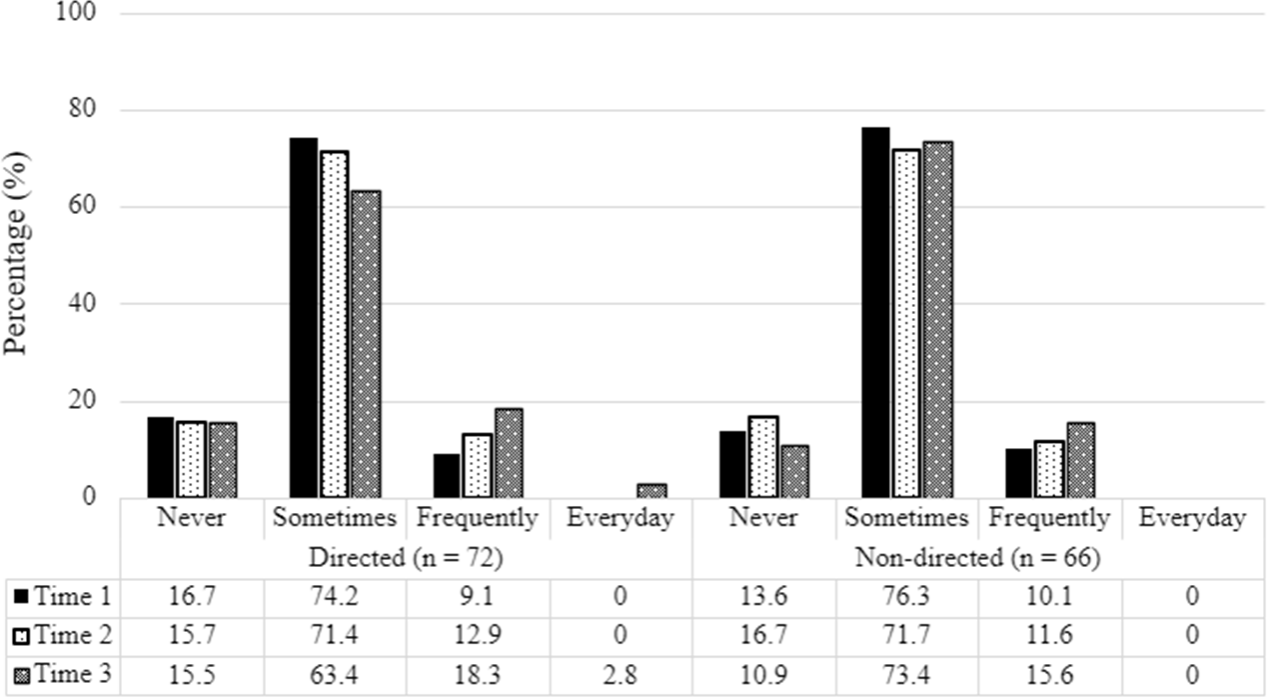
Percentage of participants reporting strategy use frequency in directed and nondirected groups, illustrating adherence to strategy use across Time 1, Time 2, and Time 3.

Given the importance of strategy and resource use for the accurate assessment of acceptability and effectiveness (Figure 2), the same analyses were repeated among the subsample of participants who reported using the strategies presented in the web-based resource at least sometimes across all 3 timepoints (baseline to follow-up). Results revealed no statistically significant group by time interaction for overall satisfaction; *F*_1.696,144.162_=0.266, *P*=.73, *η_p_^2^*=.003, strategy use; *F*_1.610,135.257_=0.479, *P*=.58, *η_p_^2^*=.006, planned strategy use; *F*_2,168_=1.810, *P*=.17, *η_p_^2^*=.021, and perceived impact on well-being; *F*_2,170_=0.665, *P*=.51, *η_p_^2^*=.008 (Table 4). Examination of main effects revealed no significant changes in strategy use over time for both groups using the Bonferroni correction; *F*_1.793,135.257_=0.479, *P*=.04, *η_p_^2^*=.039. Impact on well-being also did not change over time for both the directed and nondirected groups; *F*_2,170_=5.299, *P*=.01, *η_p_^2^*=.059. Overall, contrary to the first hypothesis (H1), the directed and nondirected groups did not differ in terms of overall resource acceptability, strategy use, plan for strategy use, and reported impact of strategy use on well-being.

### Objective 2: Effectiveness of the Self-Directed Digital Resource as Assessed by Group Differences (Directed Vs Nondirected Vs Comparison) Over Time (Baseline, Post, and Follow-Up) on Stress, Coping, and Well-Being Outcomes

**Figure 3.**
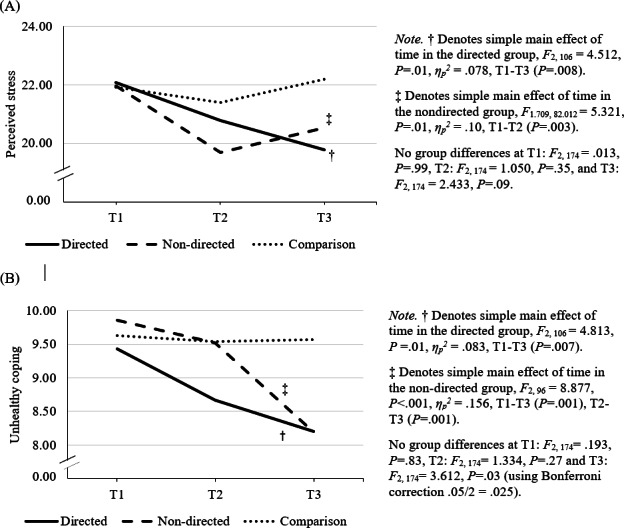
Scores on perceived stress and unhealthy coping by time and group (directed, nondirected, and comparison), depicting simple main effects and pairwise comparisons for each outcome.

A series of 2-way mixed design ANOVAs was conducted to assess group (directed, nondirected, and comparison) by time (baseline; T1, post; T2, follow-up; T3) interactions for stress, coping (coping self-efficacy, healthy coping, unhealthy coping behaviors), and well-being outcomes. As depicted in Table 2 and Figure 3, results revealed significant group-by-time interactions for stress and unhealthy coping; however, no significant interactions were found for coping self-efficacy, healthy coping, or well-being. Partially supporting hypothesis H2a, the directed group demonstrated significant improvements across stress and unhealthy coping in contrast to the comparison group; however, there were no differences between the directed and nondirected groups. Hypothesis H2b pertaining to changes in stress, coping, and well-being in the directed group relative to the comparison group was also partially supported.

Examination of simple main effects of group using the Bonferroni correction revealed no differences between groups for either stress or unhealthy coping across any of the timepoints. Patterns for the simple MET indicate that stress (*P*=.01, *ƞ_p_^2^*=.078) and unhealthy coping (*P*=.01, *ƞ_p_^2^*=.10) decreased over time within both the directed and nondirected groups but stayed stable across timepoints within the comparison group (Figure 3). Specifically, the observed decrease in stress took place between T1 and T3 (*P*=.008) for the directed group, and between T1 and T2 (*P*=.003) for the nondirected group. Unhealthy coping decreased between T1 and T3 in both groups (directed: *P*=.007, nondirected: *P*=.001), and the decrease between T2 and T3 (*P*=.001) was significant for the nondirected group.

Analyses of main effects for the nonsignificant interactions revealed a significant MET for coping self-efficacy (*P*<.001, *ƞ_p_^2^*=.049) and healthy coping (*P*<.001, *ƞ_p_^2^*p2=.084) with pairwise comparisons using the Bonferroni correction revealing a significant increase in coping self-efficacy from T1 to T3 (*P*<.001). Similarly, healthy coping showed a significant increase from T1 to T2 (*P*=.001) and from T1 to T3 (*P*<.001) across all groups.

### Objective 3 (Merged Groups): Effectiveness of the Self-Directed Digital Resource as Assessed by Group Differences (Resource Group Vs Comparison) Over Time (Baseline, Post, and Follow-Up) on Stress, Coping, and Well-Being Outcomes

A series of 2-way mixed design ANOVAs was conducted to assess group (resource group; merged directed and nondirected vs comparison) by time (baseline; T1, post; T2, follow-up; T3) interactions for stress, coping (coping self-efficacy, healthy coping, unhealthy coping behaviors), and well-being outcomes. As depicted in Table 3, significant group-by-time interactions were found for stress and coping outcomes, although no interaction was detected for well-being. As expected, results revealed significant decreases in stress and unhealthy coping, as well as increases in coping self-efficacy and healthy coping among the resource group over time, in contrast to the comparison group. Thus, hypothesis H3 was partially supported, given that no changes in well-being were detected.

**Figure 4.**
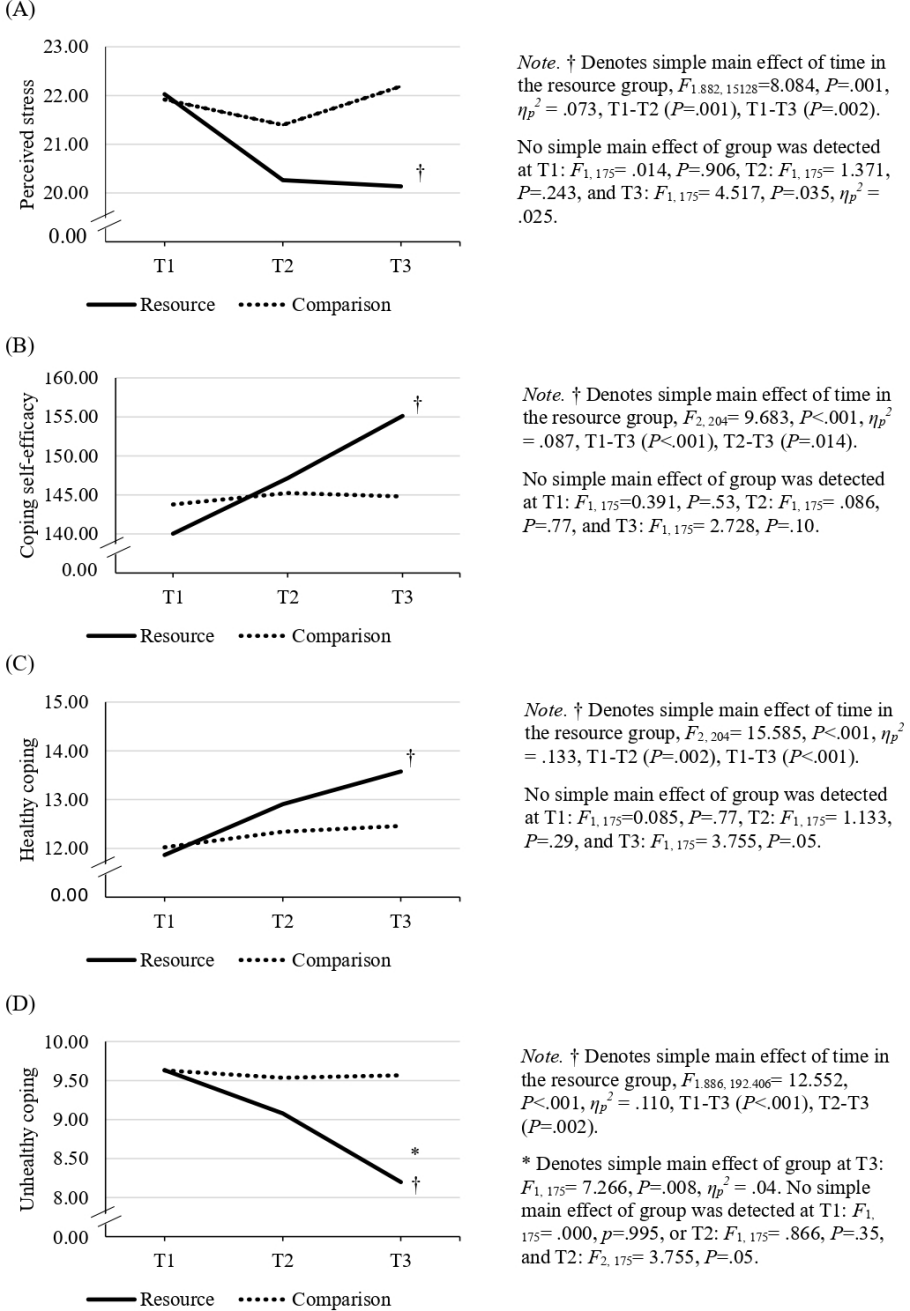
Scores on stress and coping outcomes by time and group (resource, comparison), depicting simple main effects of time and group as well as pairwise comparisons for each outcome.

Analyses of simple main effects of time and group for the outcomes of stress, coping self-efficacy, healthy, and unhealthy coping are presented in Figure 4. In terms of the simple main effects of time, the resource group showed significant decreases in stress (*P*=.001, *ƞ_p_^2^*=.073) and unhealthy coping (*P*<.001, *ƞ_p_^2^*=.110), and significant increases in coping self-efficacy (*P*<.001, *ƞ_p_^2^*=.087) and healthy coping (*P*<.001, *ƞ_p_^2^*=.133) over time, in contrast to the comparison group. The observed changes over time took place between T1 and T3 for all outcomes (Figure 4), with significant changes detected between T1 and T2 for stress (decrease; *P*=.001) and healthy coping (increase; *P*=.002). Furthermore, coping self-efficacy significantly increased (*P*=.01) and unhealthy coping decreased (*P*=.002) between T2 and T3 within the resource group. In terms of the simple main effect of group, the resource group reported significantly lower unhealthy coping (*P*=.008, *ƞ_p_^2^*=.04) at the follow-up timepoint in contrast to the comparison group; no other group differences were detected between the resource and comparison groups.

## Discussion

### Principal Findings

This study sought to examine the acceptability and effectiveness of sharing a collection of evidence-based stress-management and healthy coping strategies and multimedia resources on a website for university students’ self-directed use. Overall, students rated the resources and strategies presented on the website very highly, with comparably high rates of satisfaction reported by both those who received personalized recommendations after screening (ie, directed) and those who did not receive personalized recommendations (ie, nondirected). This finding is consistent with previous studies reporting high levels of receptivity and interest for digital, self-directed support options among university populations [32,42,68]. However, it was interesting that there was no added benefit of the screening and sharing personalized recommendations approach within this study. It is possible that high satisfaction with the overall web-based resource and the breadth of information shared constitutes a ceiling effect that prevented the detection of any unique benefits of screening in this study. This is consistent with previous findings where university students reported high levels of satisfaction with a self-directed, video outreach program [69]. These results potentially allude to students’ high receptivity to information about stress management and healthy coping that is presented in multimedia, self-paced, and visually engaging formats. Furthermore, it is possible that the use of emerging adulthood as a developmental framework and the inclusion of students as part of the project team across all stages of resource development and evaluation contributed to the creation of materials that were particularly relevant for students and were ultimately very well received.

A small proportion of students (35/242, 14.50%) reported never using the digital resource and strategies over the duration of the study. While issues with resource uptake and use were expected, given earlier research findings [36,38,51], it was encouraging that the majority of participants (77/242, 83.49%) reported at least some use of the self-directed website in this study. Exclusion of the subgroup of participants reporting no uptake did not impact the findings of acceptability, revealing comparably high levels of satisfaction across both study groups over time.

In terms of effectiveness, stress and engagement in unhealthy coping behaviors both decreased in the directed and nondirected groups, with no changes observed in the comparison group. Overall, these findings suggest that using the digital resource led to improvements in stress and unhealthy coping; however, there was no added benefit of the screening and referral approach. It is possible that screening had no impact in this study because (1) the researcher-developed measure may not have been sensitive enough to identify groups of need that were meaningfully distinct, or (2) students’ need for support was limited in variability in the study sample. If the sample included a greater proportion of students demonstrating a high need for stress management and healthy coping support, they may have benefited to a greater extent from receiving personalized resources.

Finally, the 2 resource groups (directed and nondirected) were merged to examine the effectiveness of the overall digital resource against the comparison group for the same outcomes (ie, stress, coping self-efficacy, coping behaviors, and well-being). Findings revealed significant improvements across stress and coping, although there was no effect on well-being. As hypothesized, stress and unhealthy coping decreased, whereas coping self-efficacy and healthy coping increased from baseline to follow-up among the resource group, with no changes detected in the comparison group. Additionally, the pattern of change was similar across the outcomes where changes were detected for stress and healthy coping between baseline and post timepoints, and changes for coping self-efficacy and unhealthy coping detected between post and follow-up timepoints. Contrary to what was expected, there were no changes in well-being across any of the groups over time. This finding contradicts that of Chung et al [36], who reported significant improvements in well-being (using the same measure) following students’ use of a digital self-directed mindfulness program for university students. However, the timeline between baseline and follow-up assessments was shorter in this study (10 wk) in comparison to the 14 weeks between baseline and follow-up in the study by Chung et al [36]. It is therefore possible that additional time is needed to detect changes in subjective well-being in response to engagement with self-directed programming.

Taken together, the findings support the effectiveness of sharing stress-management and healthy coping resources on a self-directed digital platform for improving university students’ stress and coping outcomes while demonstrating that the web-based resource was well-received. This study builds on the emerging evidence base highlighting the promise of enhancing university student stress management and coping capacity through universal, digital, self-directed supports [36,69]. Furthermore, findings demonstrate the potential value of extending low-intensity support options (ie, lowest steps within Stepped Care 2.0) [52] beyond the context of clinical service delivery to benefit students [70]. Given problems with help-seeking on campus [43,44], the integration of low-intensity, self-directed stress-management and coping support across the whole university can function to proactively connect students with evidence-based resources.

### Contributions

The unique contribution of this study towards research and practice in supporting university students’ stress management and healthy coping is threefold. First, this study contributes to the small but growing evidence base demonstrating the feasibility, acceptability, and effectiveness of low-resource, self-directed programming for supporting students’ stress and coping outcomes in demanding university environments [36,69]. Second, this study responds to calls for enhancing access to freely available and trustworthy digital resources for managing stress and coping capacity as a supplement to existing mental health services on campus [39,42,71]. Similarly, this study responds to calls to specifically promote the availability of evidence-based strategies for healthy coping in university environments to support coping capacity and mitigate the negative impacts of engaging in unhealthy coping behaviors [7,22,41]. Third, this study presented the first adaptation of the clinical screening and referral to stepped care approach for use across the general university student population to connect them with lower-intensity resources proportional to their reported level of need for stress-management and healthy coping information. While there was no evidence for a differential benefit of this adapted approach in this study, the results suggest that the screening and directing approach may vary in its effectiveness if used with those with low needs and may only be beneficial when targeting those with a more severe need for support around stress and coping. Last, the web-based, self-directed resource format tested in this study is scalable to other higher education contexts and adaptable to university student populations. The current format allows for low-resource, wide-reaching, and sustainable implementation of student stress and coping support compared with more resource-intensive formats, such as in-person or synchronous options. While the format is inherently scalable, challenges exist at the development and implementation stages, including the initial investment in material development, integration with institutional digital infrastructure, and promotion to ensure student use and engagement. Nonetheless, this upfront effort is worthwhile, as the resource offers sustainable, flexible support with demonstrated benefits for university student stress and coping.

### Limitations and Future Directions

Study findings must be interpreted with consideration of the following limitations. First, the timeline of the evaluation study was constrained to a relatively brief 10-week period. Although this timeframe allowed for a focused examination of the specific variables under consideration, it also limits the ability to capture longer-term effects or variations that could emerge over an extended period. Future studies with extended timelines are warranted to explore the sustainability and long-term impacts of web-based, self-directed resources to support university student stress management and coping capacity. Second, one of the measures used (ie, the Coping Index; CI) [64], exhibited poor internal consistency within the health and unhealthy coping subscales. While it was included in this study, given the measure’s direct alignment with the theoretical foundations of the study (ie, health theory of coping) [7], caution is advised for future uses of this measure in research in the absence of psychometric validation. Third, students identifying as women were overrepresented in the study sample, which impacts the generalizability of findings. While this is commonly observed across social science research [72], it is crucial for future studies to explore means of engaging participants who represent a more diverse range of gender identities. Fourth, the lack of impact of the screening and directing approach tested within this study could be due to the use of a researcher-developed screening questionnaire and algorithm to facilitate the directing. It is possible that the screening questionnaire was not effective in delineating low, moderate, or high need groups. Future research could consider establishing the validity and sensitivity of the screener measure ahead of examining the effectiveness of the screening and directing approach in the context of an intervention. Fifth, although the web-based resource tested in this study was designed to enhance access to stress and coping supports in the university context, it should be acknowledged that access to reliable internet, personal devices, or private spaces to engage with the content is not universal. These issues present barriers and may affect the generalizability of the findings and the scalability of the resource across diverse higher education settings. As research on digital mental health and well-being programming advances, it is essential to consider and address barriers related to digital equity to ensure broad and inclusive access to support. Finally, a notable limitation in this study is the absence of consideration of intraindividual identity factors (eg, gender, racial/ethnic identity) or lived experience (eg, history of mental illness and/or trauma). Although this study demonstrates the acceptability and effectiveness of a web-based, self-directed resource for supporting university students’ stress-management and coping capacity, what remains to be explored is the potentially differential acceptability and effectiveness of the self-directed support option as a function of intraindividual identity factors.

### Conclusions

In summary, this study highlights the acceptability and effectiveness of a self-directed, web-based resource providing evidence-based stress-management and healthy coping strategies for university students. Results indicate that students tended to like the overall resource and were satisfied with the content and format of the information presented, although there was no added benefit of the screening and directing approach in this study. Importantly, students’ engagement with the resource and use of the strategies led to improvements in stress, their belief in their capacity to cope, and their engagement in healthier coping behaviors. Thus, the web-based resource evaluated in this study demonstrates promise for supplementing existing mental health services on campus to provide additional support for managing stress and enhancing coping capacity among university students.

## Supplementary material

10.2196/74205 Multimedia Appendix 1 Supplemental file providing additional details on participants’ ratings of resource acceptability, the screening measure and algorithm, and screenshots of the web-based resource tested in this study.

10.2196/74205 Checklist 1 CONSORT-eHEALTH checklist (V 1.6.1).

## Abbreviations

CI: Coping Index
CSE: Coping Self-Efficacy
MET: main effect of time
PSS: Perceived Stress Scale
SC2.0: Stepped Care 2.0
T1: baseline
T2: post
T3: follow-up
WEMWBS: Warwick-Edinburgh Mental Well-Being Scale

## References

[1] American College Health Association (2022). National college health assessment III undergraduate student reference group executive summary. https://www.acha.org/wp-content/uploads/2024/07/NCHA-III_SPRING_2022_UNDERGRAD_REFERENCE_GROUP_EXECUTIVE_SUMMARY.pdf.

[2] American College Health Association (2022). National college health assessment III Canadian reference group executive summary. https://www.acha.org/wp-content/uploads/2024/07/NCHA-III_SPRING_2022_CANADIAN_REFERENCE_GROUP_EXECUTIVE_SUMMARY.pdf.

[3] Sharp J, Theiler S (2018). A review of psychological distress among university students: pervasiveness, implications and potential points of intervention. Int J Adv Counselling.

[4] Stallman HM (2010). Psychological distress in university students: a comparison with general population data. Aust Psychol.

[5] Harrer M, Adam SH, Fleischmann RJ (2018). Effectiveness of an internet- and app-based intervention for college students with elevated stress: randomized controlled trial. J Med Internet Res.

[6] Lattie EG, Adkins EC, Winquist N, Stiles-Shields C, Wafford QE, Graham AK (2019). Digital mental health interventions for depression, anxiety, and enhancement of psychological well-being among college students: systematic review. J Med Internet Res.

[7] Stallman HM (2020). Health theory of coping. Aust Psychol.

[8] Brown JSL (2018). Student mental health: some answers and more questions. J Ment Health.

[9] Hill M, Farrelly N, Clarke C, Cannon M (2024). Student mental health and well-being: overview and future directions. Ir J Psychol Med.

[10] Van de Velde S, Buffel V, Bracke P (2021). The COVID-19 international student well-being study. Scand J Public Health.

[11] Conley CS, Kirsch AC, Dickson DA, Bryant FB (2014). Negotiating the transition to college: developmental trajectories and gender differences in psychological functioning, cognitive-affective strategies, and social well-being. Emerg Adulthood.

[12] Arnett JJ (2000). Emerging adulthood. a theory of development from the late teens through the twenties. Am Psychol.

[13] Arnett JJ (2004). Emerging Adulthood: The Winding Road from the Late Teens Through the Twenties.

[14] Swanson JA (2016). Trends in literature about emerging adulthood: review of empirical studies. Emerg Adulthood.

[15] Syed M, Mitchell LL (2013). Race, ethnicity, and emerging adulthood: retrospect and prospects. Emerg Adulthood.

[16] Böke BN, Mills DJ, Mettler J, Heath NL (2019). Stress and coping patterns of university students. J Coll Stud Dev.

[17] Bukobza G (2009). Relations between rebelliousness, risk-taking behavior, and identity status during emerging adulthood. Identity (Mahwah, N J).

[18] Sussman S, Arnett JJ (2014). Emerging adulthood: developmental period facilitative of the addictions. Eval Health Prof.

[19] Slimmen S, Timmermans O, Mikolajczak-Degrauwe K, Oenema A (2022). How stress-related factors affect mental wellbeing of university students a cross-sectional study to explore the associations between stressors, perceived stress, and mental wellbeing. PLoS One.

[20] Cunningham S, Duffy A (2019). Investing in our future: importance of postsecondary student mental health research. Can J Psychiatry.

[21] Munthali RJ, Richardson CG, Pei J, Westenberg JN, Munro L, Auerbach RP (2023). Patterns of anxiety, depression, and substance use risk behaviors among university students in Canada. J Am Coll Health.

[22] Stallman HM, Lipson SK, Zhou S, Eisenberg D (2022). How do university students cope? an exploration of the health theory of coping in a US sample. J Am Coll Health.

[23] Amanvermez Y, Rahmadiana M, Karyotaki E (2020). Stress management interventions for college students: a systematic review and meta-analysis. Clinical Psychology: Science and Practice.

[24] Ma L, Zhang Y, Cui Z (2019). Mindfulness-based interventions for prevention of depressive symptoms in university students: a meta-analytic review. Mindfulness (N Y).

[25] Ang WHD, Lau ST, Cheng LJ (2022). Effectiveness of resilience interventions for higher education students: a meta-analysis and metaregression. J Educ Psychol.

[26] Worsley JD, Pennington A, Corcoran R (2022). Supporting mental health and wellbeing of university and college students: a systematic review of review-level evidence of interventions. PLoS One.

[27] Conley CS, Durlak JA, Kirsch AC (2015). A meta-analysis of universal mental health prevention programs for higher education students. Prev Sci.

[28] Harrer M, Adam SH, Baumeister H (2019). Internet interventions for mental health in university students: a systematic review and meta-analysis. Int J Methods Psychiatr Res.

[29] Bolinski F, Boumparis N, Kleiboer A, Cuijpers P, Ebert DD, Riper H (2020). The effect of e-mental health interventions on academic performance in university and college students: a meta-analysis of randomized controlled trials. Internet Interv.

[30] Fleming T, Bavin L, Lucassen M, Stasiak K, Hopkins S, Merry S (2018). Beyond the trial: systematic review of real-world uptake and engagement with digital self-help interventions for depression, low mood, or anxiety. J Med Internet Res.

[31] Gabrielli S, Rizzi S, Bassi G (2021). Engagement and effectiveness of a healthy-coping intervention via chatbot for university students during the COVID-19 pandemic: mixed methods proof-of-concept study. JMIR mHealth uHealth.

[32] Lattie EG, Lipson SK, Eisenberg D (2019). Technology and college student mental health: challenges and opportunities. Front Psychiatry.

[33] Fischer R, Bortolini T, Karl JA, Zilberberg M, Robinson K, Rabelo A (2020). Rapid review and meta-meta-analysis of self-guided interventions to address anxiety, depression, and stress during COVID-19 social distancing. Front Psychol.

[34] Cuijpers P, Donker T, Johansson R, Mohr DC, van Straten A, Andersson G (2011). Self-guided psychological treatment for depressive symptoms: a meta-analysis. PLoS One.

[35] Karyotaki E, Riper H, Twisk J (2017). Efficacy of self-guided internet-based cognitive behavioral therapy in the treatment of depressive symptoms. JAMA Psychiatry.

[36] Chung J, Mundy ME, McKenzie S (2022). A self-managed online mindfulness program in a university-wide learning management system orientation site: a real-world ecological validation study. Front Psychol.

[37] Fleischmann RJ, Harrer M, Zarski AC, Baumeister H, Lehr D, Ebert DD (2018). Patients’ experiences in a guided internet- and app-based stress intervention for college students: a qualitative study. Internet Interv.

[38] Lillevoll KR, Vangberg HCB, Griffiths KM, Waterloo K, Eisemann MR (2014). Uptake and adherence of a self-directed internet-based mental health intervention with tailored e-mail reminders in senior high schools in Norway. BMC Psychiatry.

[39] Becker TD, Torous JB (2019). Recent developments in digital mental health interventions for college and university students. Curr Treat Options Psych.

[40] Oti O, Pitt I (2021). Online mental health interventions designed for students in higher education: a user-centered perspective. Internet Interv.

[41] Reis A, Saheb R, Parish P, Earl A, Klupp N, Sperandei S (2021). How I cope at university: self‐directed stress management strategies of Australian students. Stress and Health.

[42] Ahuvia IL, Sung JY, Dobias ML, Nelson BD, Richmond LL, London B (2022). College student interest in teletherapy and self-guided mental health supports during the COVID-19 pandemic. J Am Coll Health.

[43] Bourdon JL, Moore AA, Long EC, Kendler KS, Dick DM (2020). The relationship between on-campus service utilization and common mental health concerns in undergraduate college students. Psychol Serv.

[44] Dunley P, Papadopoulos A (2019). Why is it so hard to get help? Barriers to help-seeking in postsecondary students struggling with mental health issues: a scoping review. Int J Ment Health Addiction.

[45] Lakhtakia T, Torous J (2022). Current directions in digital interventions for mood and anxiety disorders. Curr Opin Psychiatry.

[46] Eisenberg D, Hunt J, Speer N, Zivin K (2011). Mental health service utilization among college students in the United States. J Nerv Ment Dis.

[47] Cho S, Bastien L, Petrovic J, Böke BN, Heath NL (2024). The role of mental health stigma in university students’ satisfaction with web-based stress management resources: intervention study. JMIR Form Res.

[48] Böke BN, Joly M, Bastien L, Heath NL (2024). Keep it brief: can a 4-item stress screener predict university adjustment over 18 months?. High Educ Res Dev.

[49] Hasking PA, Robinson K, McEvoy P (2024). Development and evaluation of a predictive algorithm and telehealth intervention to reduce suicidal behavior among university students. Psychol Med.

[50] King CA, Eisenberg D, Zheng K (2015). Online suicide risk screening and intervention with college students: a pilot randomized controlled trial. J Consult Clin Psychol.

[51] Rith-Najarian LR, Boustani MM, Chorpita BF (2019). A systematic review of prevention programs targeting depression, anxiety, and stress in university students. J Affect Disord.

[52] Cornish P (2020). Stepped Care 2.0: A Paradigm Shift in Mental Health.

[53] Cornish PA, Berry G, Benton S (2017). Meeting the mental health needs of today’s college student: reinventing services through Stepped Care 2.0. Psychol Serv.

[54] (2008). Web content accessibility guidelines (WCAG). World Wide Web Consortium.

[55] Cohen S, Williamson GM, Spacapan S, Oskamp S (1988). The Social Psychology of Health.

[56] Chesney MA, Neilands TB, Chambers DB, Taylor JM, Folkman S (2006). A validity and reliability study of the coping self-efficacy scale. Br J Health Psychol.

[57] Russell D, Peplau LA, Cutrona CE (1980). The revised UCLA loneliness scale: concurrent and discriminant validity evidence. J Pers Soc Psychol.

[58] Zimet GD, Dahlem NW, Zimet SG, Farley GK (1988). The multidimensional scale of perceived social support. J Pers Assess.

[59] Armstrong S, Oomen-Early J (2009). Social connectedness, self-esteem, and depression symptomatology among collegiate athletes versus nonathletes. J Am Coll Health.

[60] Kirkpatrick JD, Kirkpatrick WK (2016). Kirkpatrick’s Four Levels of Training Evaluation.

[61] Cohen S, Kamarck T, Mermelstein R (1983). A global measure of perceived stress. J Health Soc Behav.

[62] Denovan A, Dagnall N, Dhingra K, Grogan S (2019). Evaluating the perceived stress scale among UK university students: implications for stress measurement and management. Stud High Educ.

[63] Luberto CM, Cotton S, McLeish AC, Mingione CJ, O’Bryan EM (2014). Mindfulness skills and emotion regulation: the mediating role of coping self-efficacy. Mindfulness (N Y).

[64] Stallman HM, Beaudequin D, Hermens DF, Eisenberg D (2021). Modelling the relationship between healthy and unhealthy coping strategies to understand overwhelming distress: A Bayesian network approach. Journal of Affective Disorders Reports.

[65] Stallman HM (2019). Efficacy of the My Coping Plan mobile application in reducing distress: a randomised controlled trial. Clin Psychol (Aust Psychol Soc).

[66] Meyers LS, Gamst G, Guarino AJ (2017). Applied Multivariate Research: Design and Interpretation.

[67] Tennant R, Hiller L, Fishwick R (2007). The Warwick-Edinburgh mental well-being scale (WEMWBS): development and UK validation. Health Qual Life Outcomes.

[68] Neal DM, Campbell AJ, Williams LY, Liu Y, Nussbaumer D (2011). “I did not realize so many options are available”: cognitive authority, emerging adults, and e-mental health. Lib Inf Sci Res.

[69] Bastien L, Boke BN, Mettler J (2022). Peer-presented versus mental health service provider-presented mental health outreach programs for university students: randomized controlled trial. JMIR Ment Health.

[70] Ryan ML, Shochet IM, Stallman HM (2010). Universal online interventions might engage psychologically distressed university students who are unlikely to seek formal help. Adv Ment Health.

[71] Montagni I, Tzourio C, Cousin T, Sagara JA, Bada-Alonzi J, Horgan A (2020). Mental health-related digital use by university students: a systematic review. Telemed J E Health.

[72] Becker R (2022). Gender and survey participation: an event history analysis of the gender effects of survey participation in a probability-based multi-wave panel study with a sequential mixed-mode design. Methods Data Anal.

