# Supplementary material for: Examining the Acceptability and Effectiveness of a Self-Directed, Web-Based Resource for Stress and Coping in University: Randomized Controlled Trial
Source: JMIR Ment Health. 2026 Jan 23;13:e74205. doi: 10.2196/74205 (PMC12829895; doi:10.2196/74205)

Multimedia Appendix

Table 1d

Participant ratings of acceptability (satisfaction, actual and planned strategy use, impact on well-being) among those in the directed group (n = 72).

	Group 1: Directed (N = 72)											
	Time 1				Time 2				Time 3			
	Strongly disagree	Disagree	Agree	Strongly agree	Strongly disagree	Disagree	Agree	Strongly agree	Strongly disagree	Disagree	Agree	Strongly agree
	n (%)	*n* (%)	*n* (%)	*n* (%)	*n* (%)	*n* (%)	*n* (%)	*n* (%)	*n* (%)	*n* (%)	*n* (%)	*n* (%)
**1. I found the website useful for me**	4 (6.1)	13 (19.7)	43 (65.2)	6 (9.1)	2 (2.9)	16 (22.9)	51 (72.9)	1 (1.4)	6 (8.5)	11 (15.5)	50 (70.4)	4 (5.6)
**2. I found the content in the website was presented in an engaging manner**	4 (6.1)	10 (15.2)	35 (53.0)	17 (25.8)	3 (4.3)	15 (21.4)	40 (57.1)	12 (17.1)	2 (2.8)	10 (14.1)	45 (63.4)	14 (19.7)
**3. I found that the website presented valuable strategies and techniques**	1 (1.5)	5 (7.7)	42 (64.6)	17 (26.2)	1 (1.4)	4 (5.7)	51 (72.9)	14 (20.0)	2 (2.8)	5 (7.0)	48 (67.6)	16 (22.5)
**4. I would recommend this website to other university students**	2 (3.0)	12 (18.2)	33 (50.0)	19 (28.8)	2 (2.9)	10 (14.3)	37 (52.9)	21 (30.0)	3 (4.2)	8 (11.3)	38 (53.5)	22 (31.0)
**5. The strategies presented in the website helped me better understand how to manage my stress and improve my wellness**	1 (1.5)	18 (27.3)	38 (57.6)	9 (13.6)	2 (2.9)	14 (20.0)	51 (72.9)	3 (4.3)	2 (2.8)	10 (14.1)	45 (63.4)	14 (19.7)
**6. The strategies presented in the website were easy to understand**	3 (4.5)	2 (3.0)	41 (62.1)	20 (30.3)	1 (1.4)	6 (8.7)	39 (56.5)	23 (33.3)	0 (0)	5 (7.0)	38 (53.5)	28 (39.4)
**7. I feel confident in my understanding of the suggested strategies on the website**	4 (6.1)	4 (6.1)	37 (56.1)	21 (31.8)	1 (1.4)	3 (4.3)	45 (65.2)	20 (29.0)	0 (0)	6 (8.5)	42 (59.2)	23 (32.4)
**8. The website has motivated me to try out these strategies**	6 (9.1)	19 (28.8)	33 (50.0)	8 (12.1)	3 (4.3)	21 (30.0)	38 (54.3)	8 (11.4)	5 (7.1)	12 (17.1)	40 (57.1)	13 (18.6)
	Never	Sometimes	Frequently	Everyday	Never	Sometimes	Frequently	Everyday	Never	Sometimes	Frequently	Everyday
**Over the past two weeks, how often did you use the strategies presented on the website?**	11 (16.7)	49 (74.2)	6 (9.1)	0 (0)	11 (15.7)	50 (71.4)	9 (12.9)	0 (0)	11 (15.5)	45 (63.4)	13 (18.3)	2 (2.8)
**Over the coming weeks, I plan to use the strategies presented on the website**	4 (6.1)	41 (62.1)	20 (30.3)	1 (1.5)	2 (2.9)	41 (59.4)	20 (29.0)	6 (8.7)	7 (9.9)	41 (57.7)	20 (28.2)	3 (4.2)
	No impact	Low impact	Somewhat impacted	Highly impacted	No impact	Low impact	Somewhat impacted	Highly impacted	No impact	Low impact	Somewhat impacted	Highly impacted
**Over the past two weeks, how would you rate the impact of the strategies presented on the website on your well-being?**	10 (15.2)	19 (28.8)	33 (50.0)	4 (6.1)	8 (11.4)	18 (25.7)	40 (57.1)	4 (5.7)	9 (12.7)	10 (14.1)	46 (64.8)	6 (8.5)

Note. Different *n* between timepoints is a result of participant attrition over time.

Multimedia Appendix

Table 1e

Participant ratings of acceptability (satisfaction, actual and planned strategy use, impact on well-being) among those in the non-directed group (n = 66).

	Group 2: Non-directed (N = 66)											
	Time 1				Time 2				Time 3			
	Strongly disagree	Disagree	Agree	Strongly agree	Strongly disagree	Disagree	Agree	Strongly agree	Strongly disagree	Disagree	Agree	Strongly agree
	n (%)	*n* (%)	*n* (%)	*n* (%)	*n* (%)	*n* (%)	*n* (%)	*n* (%)	*n* (%)	*n* (%)	*n* (%)	*n* (%)
**1. I found the website useful for me**	0 (0)	10 (16.9)	46 (78.0)	3 (5.1)	1 (1.6)	13 (21.3)	46 (75.4)	1 (1.6)	2 (3.1)	12 (18.8)	46 (71.9)	4 (6.3)
**2. I found the content in the website was presented in an engaging manner**	0 (0)	10 (16.9)	40 (67.8)	9 (15.3)	2 (3.3)	9 (14.8)	45 (73.8)	5 (8.2)	2 (3.1)	7 (10.9)	46 (71.9)	9 (14.1)
**3. I found that the website presented valuable strategies and techniques**	0 (0)	5 (8.5)	45 (76.3)	9 (15.3)	0 (0)	2 (3.3)	50 (82.0)	9 (14.8)	1 (1.6)	4 (6.3)	49 (76.6)	10 (15.6)
**4. I would recommend this website to other university students**	1 (1.7)	10 (16.9)	38 (64.4)	10 (16.9)	0 (0)	10 (16.4)	34 (55.7)	17 (27.9)	1 (1.6)	7 (10.9)	37 (57.8)	19 (29.7)
**5. The strategies presented in the website helped me better understand how to manage my stress and improve my wellness**	1 (1.7)	10 (16.9)	40 (67.8)	8 (13.6)	1 (1.6)	9 (14.8)	43 (70.5)	8 (13.1)	2 (3.2)	11 (17.5)	39 (61.9)	11 (17.5)
**6. The strategies presented in the website were easy to understand**	1 (1.7)	3 (5.1)	38 (64.4)	17 (28.8)	0 (0)	7 (11.5)	38 (62.3)	16 (26.2)	1 (1.6)	3 (4.7)	39 (60.9)	21 (32.8)
**7. I feel confident in my understanding of the suggested strategies on the website**	0 (0)	11 (18.6)	36 (61.0)	12 (20.3)	0 (0)	7 (11.5)	44 (72.1)	10 (16.4)	1 (1.6)	10 (15.6)	41 (64.1)	12 (18.8)
**8. The website has motivated me to try out these strategies**	0 (0)	16 (27.6)	36 (62.1)	6 (10.3)	3 (5.0)	16 (26.7)	34 (56.7)	7 (11.7)	2 (3.1)	19 (29.7)	32 (50.0)	11 (17.2)
	**Never**	**Sometimes**	**Frequently**	**Everyday**	**Never**	**Sometimes**	**Frequently**	**Everyday**	**Never**	**Sometimes**	**Frequently**	**Everyday**
**Over the past two weeks, how often did you use the strategies presented on the website?**	8 (13.6)	45 (76.3)	6 (10.2)	0 (0)	10 (16.7)	43 (71.7)	7 (11.7)	0 (0)	7 (10.9)	47 (73.4)	10 (15.6)	0 (0)
**Over the coming weeks, I plan to use the strategies presented on the website**	2 (3.4)	34 (57.6)	20 (33.9)	3 (5.1)	2 (3.0)	36 (59.0)	22 (36.1)	1 (1.6)	4 (6.3)	40 (62.5)	18 (28.1)	2 (3.1)
	**No impact**	**Low impact**	**Somewhat impacted**	**Highly impacted**	**No impact**	**Low impact**	**Somewhat impacted**	**Highly impacted**	**No impact**	**Low impact**	**Somewhat impacted**	**Highly impacted**
**Over the past two weeks, how would you rate the impact of the strategies presented on the website on your well-being?**	8 (13.6)	21 (35.6)	29 (49.2)	1 (1.7)	9 (14.8)	16 (26.2)	34 (55.7)	2 (3.3)	7 (10.9)	18 (28.1)	37 (57.8)	2 (3.1)

Note. Different *n* between timepoints is a result of participant attrition over time.

Multimedia Appendix

Table 1f

Longitudinal Correlations Between the Study Variables

	1	2	3	4	5	6	7	8	9	10	11	12	13	14	15
1. PSS T1	-														
2. CSE T1	-.516***	-													
3. CIh T1	-.225**	.547***	-												
4. CIUh T1	.443***	-.431***	-.184*	-											
5. WB T1	-.630***	.673***	.424***	-.406***	-										
6. PSS T2	.641***	-.359***	-.132**	.364***	-.450***	-									
7. CSE T2	-.397**	.682***	.307***	-.350***	.558***	-.495***	-								
8. CIh T2	-.198**	.454***	.633***	-.137**	.407***	-.246***	.441***	-							
9. CIUh T2	.371***	-.390***	-.151*	.645***	-.320***	.406***	-.500***	-.172*	-						
10. WB T2	-.553***	.564***	.260***	-.446***	.741***	-.678***	.720***	.436***	-.479***	-					
11. PSS T3	.551***	-.328***	-.176*	.352***	-.427***	.713***	-.470***	-.264***	.422***	-.560***	-				
12. CSE T3	-.306***	.630***	.367***	-.256***	.448***	-.379***	.750***	.428***	-.428***	.540***	-.522***	-			
13. CIh T3	-.134**	.441***	.628***	-.130**	.347***	-.162*	.427***	.690***	-.126**	.298***	-.262***	.518***	-		
14. CIUh T3	.290***	-.225**	-.041	.508***	-.235**	.345***	-.397***	-.071	.667***	-.395***	.452***	-.380***	-.012	-	
15. WB T3	-.464***	.460***	.289***	-.318***	.671***	-.559***	.600***	.362***	-.386***	.759***	-.701***	.661***	-.386***	-.395***	-

Note. PSS = Perceived Stress Scale; CSE = Coping Self-Efficacy; Cih = Coping Index Healthy Coping; CIUh = Coping Index Unhealthy Coping; WB = Well-being. Correlations shown for the subsample of participants used across main analyses (n = 177). ***p < .001.**p <.01.*p < .05.

Multimedia Appendix

Screener Questionnaire

Table 2a

Properties of the Researcher Developed Screener Questionnaire by Section

**Screener Section**	**Number of items**	**Range of possible scores**	**Cut-off score**	**Cronbach's alpha**	**Section development**
Stress and Coping Behaviours	6	9 - 45	36	0.465	Researcher-developed
Perceived Stress	4	0 - 16	12	0.783	Perceived Stress Scale - 4 item (Cohen et al., 1984)
Coping Self-efficacy	4	0 - 40	6	0.702	Adapted - Coping Self-Efficacy Scale (Chesney et al., 2006)
Loneliness	3	3 - 12	10	0.809	UCLA Loneliness Scale - Revised (Russell et al., 1980)
Social Support	3	3 - 21	10	0.622	Adapted - Multidimensional Scale of Perceived Social Support (Zimet et al., 1988)
Social Connectedness	4	4 - 24	8	0.839	Adapted - Social Connectedness Scale - Revised (Armstrong & Oomen-Early, 2009)

Note. Cut-off scores were calculated to correspond to the top/bottom 15th percentile of possible scores with reference to population data for each section of the screener.

Multimedia Appendix

Table 2b

Screener Algorithm to Facilitate Directing to Personalized Resources and Resource Recommendations for each Level of Need

**Low**	**Moderate**	**High**
Scores indicate student is well-positioned to cope with stress and demonstrates low need for support<br><br>Stress and Coping Behaviours < 36<br>*AND*<br>Perceived Stress < 12<br>*AND*<br>Coping Self-Efficacy > 6<br>*AND*<br>Loneliness < 10<br>*AND*<br>Social Support > 10<br>*AND*<br>Social Connectedness > 8	Scores indicate difficulty in some areas assessed by screener, demonstrates moderate need for support<br><br>Stress and Coping Behaviours ≥ 36<br>*OR*<br>Perceived Stress ≥ 12<br>*OR*<br>Coping Self-efficacy ≤ 6<br>*OR*<br>Loneliness ≥ 10<br>*OR*<br>Social Support ≤ 10<br>*OR*<br>Social Connectedness ≤ 8	Scores indicate difficulties across multiple areas assessed by screener (general, interpersonal, and intrapersonal), demonstrates high need for support<br><br>Stress and Coping Behaviours ≥ 36<br>*AND*<br>Perceived Stress ≥ 12<br>*OR*<br>Coping Self-efficacy ≤ 6<br>*AND*<br>Loneliness ≥ 10<br>*OR*<br>Social Support ≤ 10<br>*OR*<br>Social Connectedness ≤ 8
**Resource Recommendations**		
**1.** Understanding (psychoeducation)<br><br>**2.** Strategy practice postcards	**1.** Understanding (psychoeducation)<br><br>**2.** Self-directed resources on website:<br>*Managing Stress*<br>*Enhancing Performance*<br>*Well-Being*<br>*Adulting*<br>*Socializing*	**1.** Self-directed resources on website<br><br>**2.** Resources for formal support<br>*Student Services*<br>*Community Resources*<br>*Helplines*<br><br>**3.** Tips for seeking formal support

Note. Directing to personalized resources was achieved by creating three unique pages on the website for each level of need (low, moderate, high). Participants in the directed group were automatically directed to one of these three pages based on their scores on the screener. The directing process was automated through the survey platform used in the present study (Qualtrics).

Multimedia Appendix

Web-based Resource – Home Page

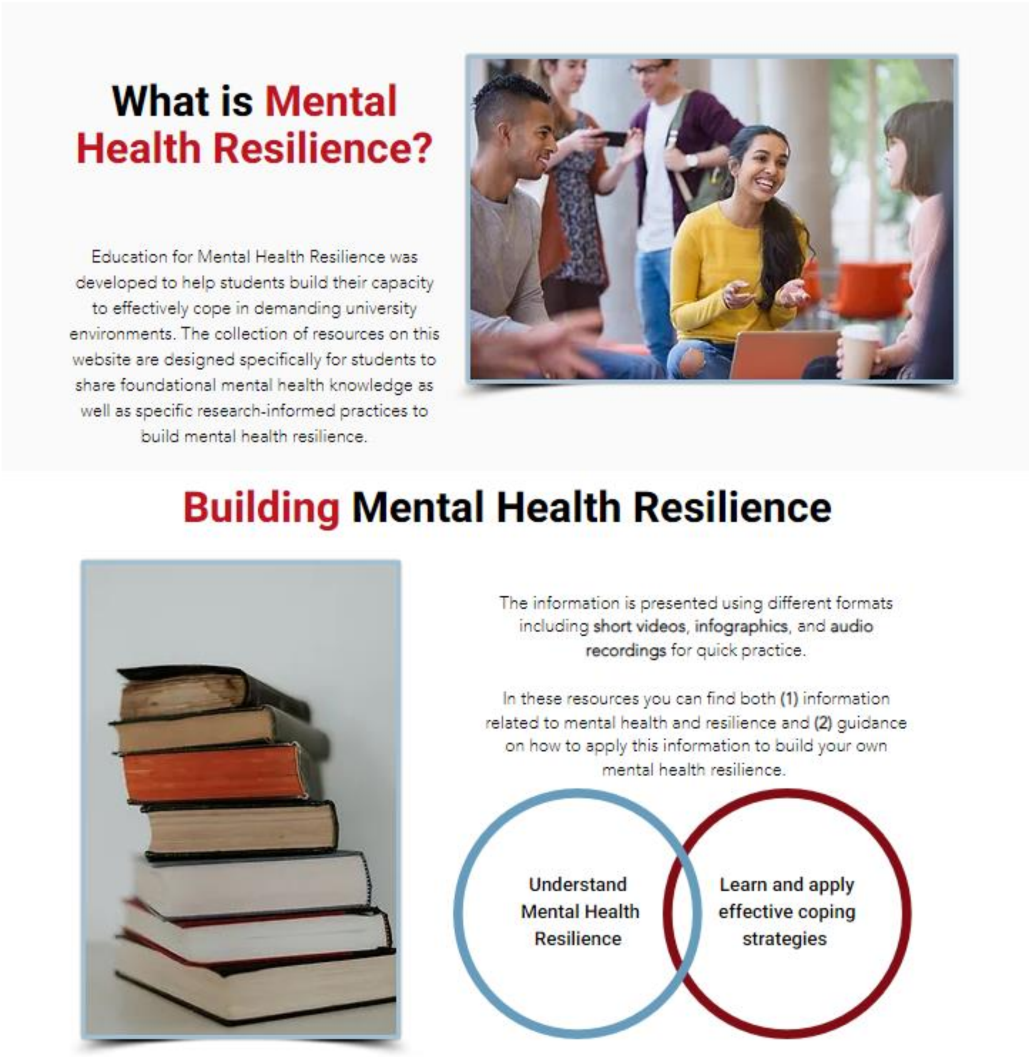

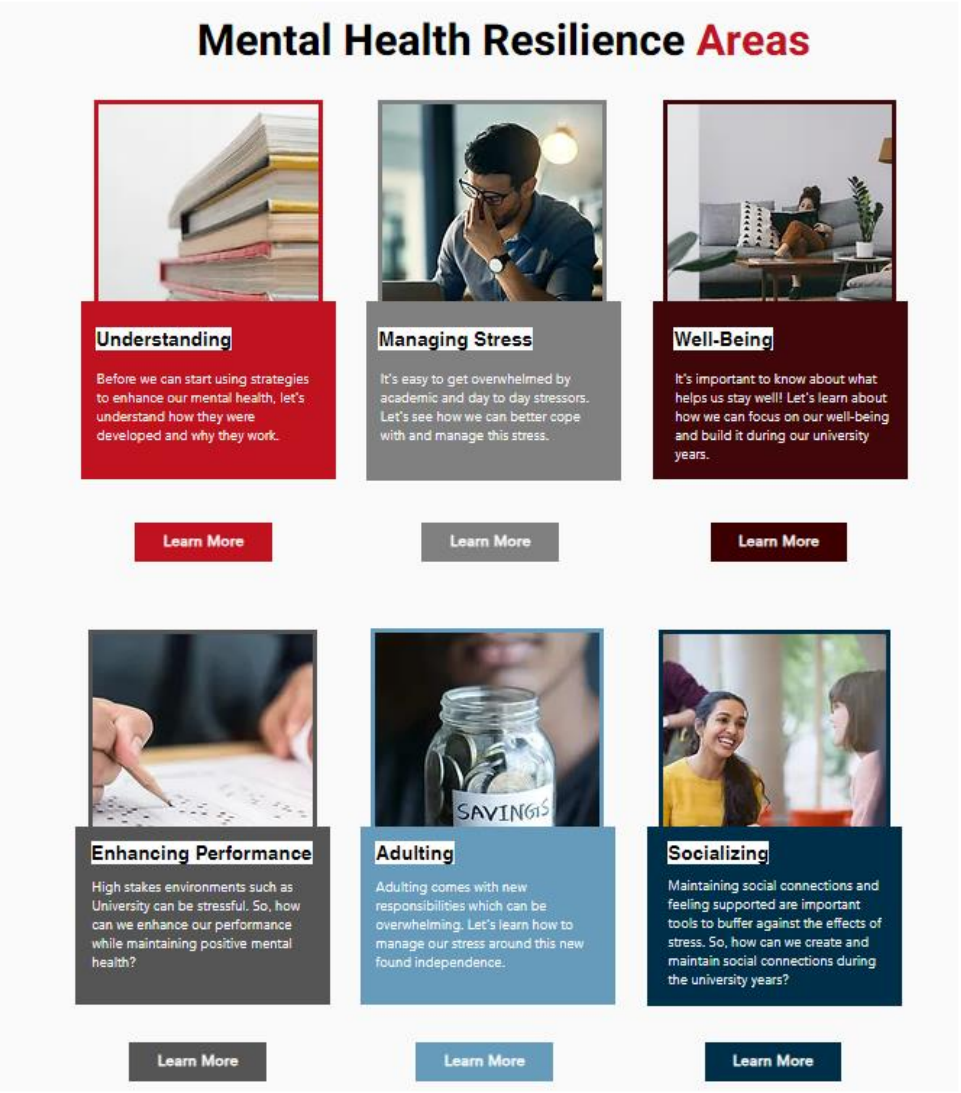

Supplement: Multimedia Appendix 1 [file mental-v13-e74205-s001.pdf]
